# A Web-Based Delphi Study for Eliciting Helpful Criteria in the Positive Diagnosis of Hemophagocytic Syndrome in Adult Patients

**DOI:** 10.1371/journal.pone.0094024

**Published:** 2014-04-07

**Authors:** Gilles Hejblum, Olivier Lambotte, Lionel Galicier, Paul Coppo, Christophe Marzac, Cédric Aumont, Laurence Fardet

**Affiliations:** 1 INSERM, UMR_S 1136, Institut Pierre Louis d'Epidémiologie et de Santé Publique, Paris, France; 2 Sorbonne Universités, UPMC Univ Paris 06, UMR_S 1136, Institut Pierre Louis d'Epidémiologie et de Santé Publique, Paris, France; 3 AP–HP, Hôpital Saint-Antoine, Unité de Santé Publique, Paris, France; 4 AP–HP, Hôpital de Bicêtre, Service de Médecine Interne, Le Kremlin Bicêtre, France; 5 Université Paris-Sud 11, Faculté de Médecine Paris-Sud 11, Le Kremlin Bicêtre, France; 6 AP–HP, Hôpital Saint-Louis, Service d'Immunologie Clinique, Paris, France; 7 AP–HP, Hôpital Saint-Antoine, Service d'Hématologie, Paris, France; 8 Sorbonne Universités, UPMC Univ Paris 06, Faculté de Médecine Pierre et Marie Curie, Paris, France; 9 AP–HP, Hôpital Saint-Antoine, Service d'Hématologie Biologique, Paris, France; 10 Sorbonne Universités, UPMC Univ Paris 06, Groupe de Recherche Clinique n°7 MyPAC, Paris, France; 11 AP–HP, Hôpital de Bicêtre, Service d'Hématologie Biologique, Le Kremlin Bicêtre, France; 12 AP–HP, Hôpital Saint-Antoine, Service de Médecine Interne, Paris, France; University of Catania, Italy

## Abstract

The diagnosis of the reactive form of hemophagocytic syndrome in adults remains particularly difficult since none of the clinical or laboratory manifestations are specific. We undertook a study in order to elicit which features constitute helpful criteria for a positive diagnosis. In this Delphi study, the features investigated in the questionnaire and the experts invited to participate in the survey were issued from a bibliographic search. The questionnaire was iteratively proposed to experts via a web-based application with a feedback of the results observed at the preceding Delphi round. Experts were asked to label each investigated criterion in one of the following categories: absolutely required, important, of minor interest, or not assessable in the routine practice environment. A positive consensus was a priori defined as at least 75% answers observed in the categories absolutely required and important. The questionnaire investigated 26 criteria and 24 experts originating from 13 countries participated in the second and final Delphi round. A positive consensus was reached for the nine following criteria: unilineage cytopenia, bicytopenia, pancytopenia, presence of hemophagocytosis pictures on a bone marrow aspirate or on a tissue biopsy, high ferritin level, fever, organomegaly, presence of a predisposing underlying disease, and high level of lactate dehydrogenase. A negative consensus was reached for 13 criteria, and an absence of consensus was observed for 4 criteria. The study constitutes the first initiative to date for defining international guidelines devoted to the positive diagnosis of the reactive form of hemophagocytic syndrome.

## Introduction

Hemophagocytic Syndrome (HS) is a severe clinical syndrome involving a defect in CD8 T cell and NK cytotoxicity leading to uncontrolled CD8 T cell and macrophage activation with a highly activated but ineffective immune response [1]–[4]. There are two distinct forms of HS. The primary form is genetic with an onset of the disease in early childhood in 70–80% of the cases. Based on a single report concerning Sweden, incidence of the primary form is estimated at 1.2 cases per year and per million children [5]. The secondary form, often referred to as the reactive form, may occur at any age, in association with infections, malignancies or autoimmune disease, commonly in immunocompromised patients [6], [7].

The diagnosis of HS in adults remains particularly difficult since none of the clinical or laboratory manifestations are specific: on the one hand, the same manifestations can be caused by the disorders that trigger HS, such as sepsis or lymphoma [8]; on the other hand, patients with HS may fail to meet a given diagnostic criterion, depending on the course of their disease when they are under examination [9]. Indeed, symptoms and biologic abnormalities can occur progressively along the time leading to diagnostic challenge at a given time. In addition, although sets of diagnosis criteria have been previously proposed [10]–[14], the weight of each criterion within a set is unknown and the proposed cut-off values have been empirically defined. All in all, no validated diagnostic guidelines for HS in adults are available to date, and the generalization of the diagnostic value for a given feature potentially reported in a given case series remains questionable.

In order to get more insight on this issue, we undertook an international Delphi survey among experts of the field for eliciting which criteria should be considered for the positive diagnosis of HS in adults. The Delphi technique [15]–[17] is a consensus method particularly attractive in contexts where only scarce data are available for guiding which response would be optimal for the given problem addressed. The Delphi process involves the anonymous completion of a questionnaire on several occasions referred to as the Delphi rounds. The process ensures the independence of the participants, allows participants to change their opinion based on detailed feedback about the responses to the previous round, and enables the convergence analysis of the response distribution.

Based on the criteria mentioned in the literature, the aim of the study was to elicit three types of criteria: those resulting in a consensus about their helpfulness, those resulting in a consensus about their non-helpfulness, and finally those for which the helpfulness remains uncertain (i.e., absence of consensus). The categorization of the features issued from the study results may contribute to improve diagnosis of reactive HS in adult patients–especially in settings where the resources are scarce in terms of knowledge and experience about HS in adults–and, as a consequence, reduce the delay for appropriate treatment. The current study may be viewed as a significant initial step towards a proposal of international guidelines for the positive diagnosis of HS in adults.

## Methods

### Devising the questionnaire

The questionnaire was based on the analysis of the scientific literature related to the criteria reported in association with HS. Using the Medline database (via Pubmed), on September 17, 2012, we selected papers published in English or French with any of the following terms: (hemophagocytic syndrome) OR (macrophage activation syndrome) OR (hemophagocytic lymphohistiocytosis) OR (hemophagocytosis). Focusing on adult cases, we excluded all publications related to pediatry literature. Papers focusing on basic science research were also excluded. The remaining publications were screened by two of us (LF and LG) and led to the selection of 26 clinical, biological or cytological criteria reported to be associated with the diagnosis of HS (Table 1). The questionnaire explored experts' opinions on the helpfulness of these 26 criteria in the positive diagnosis of a reactive HS in adults. As the aim of the study was to elicit the potential global helpfulness of these criteria in the positive diagnosis of a reactive HS in adults, we intentionally did not mention a threshold value for criteria concerning a quantitative measure (e.g., ferritin level). Instead, we used a generic form such as “high level” or “low level” in order to assess experts' opinion. The same search was used for selecting the experts that were solicited for participating in the survey. We retained the corresponding authors of the selected articles, excluding case report papers. This process resulted in a list of 63 experts for which an active email address was available, and to which an invitation to participate in the Delphi study was emailed.

**Table 1:** The study Questionnaire.

Item number: Criterion explored	Item wording*
1: Predisposing underlying disease	a predisposing underlying disease (e.g., lupus erythematosus, HIV, Still disease, lymphoma, drug-induced immunodepression) is:
2: Fever	fever is:
3: Organ failure	the presence of an organ failure (e.g. renal insufficiency, cardiac failure, respiratory distress syndrome) is:
4: Organomegaly	organomegaly (i.e., hepatomegaly, splenomegaly and/or adenomegaly) is:
5: Maculo-papular cutaneous rash	the presence of a maculo-papular cutaneous rash is:
6: Unilineage cytopenia	unilineage cytopenia (i.e. anemia or thrombocytopenia or leucopenia) is:
7: Bicytopenia	bicytopenia (e.g. anemia + thrombocytopenia, leucopenia + thrombocytopenia, anemia + leucopenia) is:
8: Pancytopenia	pancytopenia is:
9: Ferritin level	a high ferritin level is:
10: Percentage of glycosylated ferritin	a low percentage of glycosylated ferritin is:
11: Levels of transaminases	high levels of transaminases (i.e. SGOT, SGPT) are:
12: Bilirubin	a high level of total bilirubin is:
13: Gamma glutamyl transferase	a high level of gamma glutamyl transferase is:
14: Hyponatremia	hyponatremia is:
15: Fibrinogen	a low fibrinogen level is:
16: Triglycerides	a high triglyceride level is:
17: Lactate dehydrogenase	a high level of lactate dehydrogenase is:
18: Serum interleukin-2	a high level of serum interleukin-2 is:
19: Soluble CD163	a high level of soluble CD163 is:
20: Soluble CD25	a high level of soluble CD25 is:
21: Natural killer cells	a low activity of natural killer cells is:
22: Serum albumin	a low level of serum albumin is:
23: Activated partial thromboplastin time	a short activated partial thromboplastin time is:
24: C-reactive protein	a high level of C-reactive protein is:
25: D-dimer	a high level of D-dimer is:
26: Hemophagocytosis pictures	hemophagocytosis pictures on a bone marrow aspirate or tissue biopsy are:

*For all items, wording began with “For the positive diagnosis of reactive hemophagocytic syndrome,”.

In order to facilitate the completion of the survey, the 26 question items were all designed with the same pattern. Moreover, instructions for completing the survey were repeated to the experts in the web application before initiating completion at each Delphi round (see Figure S1): for each of the 26 items, the expert had to make a single choice among five following proposed answers (i.e. radio buttons) that were defined as follows:

- *absolutely required*: the absence of the criterion would make the diagnosis of HS very unlikely.

- *important*: the absence of the criterion would not exclude the possibility of HS but its presence clearly strengthens the diagnosis.

- *of minor interest*: the presence of the criterion may help you in diagnosing HS, but the absence of the criterion does not influence much your diagnosis.

- *useless*: you do not mind the presence or absence of the criterion for diagnosing HS.

- *not assessable in my routine practice environment*: Whatever the potential helpfulness of the criterion in the diagnosis, it is never assessed in your clinical practice, either because other criteria are sufficient for the diagnosis of HS or because it is not technically possible to assess this criterion in your department/hospital. (n.b. the first five items of the questionnaire did not propose this choice).

### Consensus definition

Based on the distribution of the collected answers at the last Delphi round, the rules for defining consensus were defined prior to launching the survey as follows: whenever the addition of “absolutely required” and “important” answers represented at least 75% of the answers for a given question item, we considered that there was a consensus for retaining the corresponding criterion as being helpful for a positive diagnosis. Conversely, whenever the addition of “of minor interest” and “useless” answers represented at least 75% of the answers for a given question item, we considered that there was a consensus for excluding the corresponding criterion as being helpful for a positive diagnosis. Such a consensus for excluding the criterion as being helpful was also applied to any item for which at least 50% of the answers were “not assessable”. In any other case, the corresponding criterion was considered to have an absence of consensus.

### The web-based Delphi application and process

The survey was deployed using a previously developed PHP/MySQL-based computer application devoted to the conduction of generic Delphi surveys via the Internet. This application was developed in our laboratory and has been already applied in different medical domains such as the prescription of chest X-rays in the intensive care unit [18] or expert-based determinations of the start and the end of influenza epidemics [19]. There are no universal optimal criteria defined for devising the number of rounds required in a Delphi process. On the one hand, the process can be viewed as a convergence process that should be ended when there are very limited changes between the current round and the preceding one. On the other hand, such a rationale would imply numerous rounds, which in turn might result in a progressive disaffection of participating experts, yielding a reduced value of the expertise at the final round. In the present study, at the end of each Delphi round, for each of the 26 questions, the study scientific committee was provided with a graph comparing the distributions of the answer at the current round and at the preceding round. Graphs were provided in a random order to the committee who was also blind at which question actually corresponded to each graph. After examination of the whole graph set, the committee balanced the potential advantages and disadvantages of a supplementary Delphi round, and decided whether to stop the survey or to undertake a supplementary round.

## Results

### The Delphi process

The first Delphi round period began on October 30 and ended on November 15, 2012. Of the 63 experts solicited for participation in the survey, two experts formally declined, and 26 of the remaining 61 experts (43%) completed the first round. The remaining 35 experts didn't respond to the invitation. The second Delphi round period began on November 16 and ended on December 3, 2012: the 26 experts that participated in the initial round were invited to participate in this second round and among these, 24 completed the questionnaire at the second round (see a typical completion screenshot in Figure S2). The study committee stopped the Delphi process at the second round, estimating that supplementary rounds were likely not to substantially change final results (see Figure S3), while a substantial disaffection of experts might occur. The 13 countries corresponding to the 24 experts' email addresses/institutions were Belgium, Canada, China, Denmark, France, Germany, Hong-Kong, India, Italy, Japan, Switzerland, U.S.A., and Venezuela.

### Consensus results

The distribution of the answers collected from the 24 experts at the second and final round are shown in Table 2 (the complete data set of the study is in Table S1). When applying the consensus rules *a priori* defined (see material and methods) a consensus for retaining a given criterion corresponded to 9 cases: unilineage cytopenia, bicytopenia, pancytopenia, presence of hemophagocytosis pictures on a bone marrow aspirate or on a tissue biopsy, high ferritin level, fever, organomegaly, presence of a predisposing underlying disease, and high level of lactate dehydrogenase (LDH). Conversely, a consensus for excluding a given criterion corresponded to 13 cases, with nine criteria judged useless or of limited interest (i.e., high level of D dimer, presence of a maculo papular cutaneous rash, high level of gamma glutamyl transferase, short activated partial thromboplastin time, low level of serum albumin, high level of bilirubin, hyponatremia, high level of C reactive protein, and presence of an organ failure), while the four remaining (i.e., high level of soluble CD163, low activity of natural killer cells, high level of serum interleukin 2, and high level of soluble CD25), were judged as not frequently enough assessed in routine practice for being considered. In the end, an absence of consensus was obtained for four criteria: low fibrinogen level, high triglyceride level, high levels of transaminases, and low percentage of glycosylated ferritin.

**Table 2:** Distribution of the answers at the second and last Delphi round.

	Number of answers (percentage)						
Criterion explored (type of consensus)	A*	B*	C*	D*	E*	A or B*	C or D*
Unilineage cytopenia (helpful criterion)	20 (83)	4 (17)	0 (0)	0 (0)	0 (0)	24 (100)	0 (0)
Hemophagocytosis pictures (helpful criterion)	6 (25)	18 (75)	0 (0)	0 (0)	0 (0)	24 (100)	0 (0)
Ferritin level (helpful criterion)	19 (79)	4 (17)	1 (4)	0 (0)	0 (0)	23 (96)	1 (4)
Fever (helpful criterion)	10 (42)	13 (54)	1 (4)	0 (0)	‡	23 (96)	1 (4)
Bicytopenia (helpful criterion)	7 (29)	16 (67)	1 (4)	0 (0)	0 (0)	23 (96)	1 (4)
Organomegaly (helpful criterion)	0 (0)	22 (92)	1 (4)	1 (04)	‡	22 (92)	2 (8)
Predisposing underlying disease (helpful criterion)	3 (13)	16 (67)	4 (17)	1 (04)	‡	19 (79)	
Pancytopenia (helpful criterion)	0 (0)	19 (79)	5 (21)	0 (0)	0 (0)	19 (79)	5 (21)
Lactate dehydrogenase (helpful criterion)	1 (4)	17 (71)	6 (25)	0 (0)	0 (0)	18 (75)	6 (25)
D dimer (unhelpful criterion)	0 (0)	0 (0)	15 (63)	9 (38)	0 (0)	0 (0)	24 (100)
Maculo papular cutaneous rash (unhelpful criterion)	0 (0)	0 (0)	19 (79)	5 (21)	‡	0 (0)	24 (100)
Gamma glutamyl transferase (unhelpful criterion)	0 (0)	1 (4)	18 (75)	5 (21)	0 (0)	1 (4)	23 (96)
Activated partial thromboplastin time (unhelpful criterion)	0 (0)	1 (4)	21 (88)	2 (08)	0 (0)	1 (4)	23 (96)
Serum albumin (unhelpful criterion)	0 (0)	2 (8)	14 (58)	8 (33)	0 (0)	2 (8)	22 (92)
Bilirubin (unhelpful criterion)	0 (0)	2 (8)	17 (71)	5 (21)	0 (0)	2 (8)	22 (92)
Hyponatremia (unhelpful criterion)	0 (0)	4 (17)	10 (42)	10 (42)	0 (0)	4 (17)	20 (83)
C reactive protein (unhelpful criterion)	2 (8)	3 (13)	14 (58)	5 (21)	0 (0)	5 (21)	19 (79)
Organ failure (unhelpful criterion)	1 (4)	5 (21)	17 (71)	1 (4)	‡	6 (25)	18 (75)
Soluble CD163 (unhelpful criterion)	1 (4)	6 (25)	0 (0)	0 (0)	17 (71)	7 (29)	0 (0)
Natural killer cells (unhelpful criterion)	1 (4)	5 (21)	4 (17)	0 (0)	14 (58)	6 (25)	4 (17)
Serum interleukin 2 (unhelpful criterion)	1 (4)	7 (29)	2 (8)	1 (4)	13 (54)	8 (33)	3 (13)
Soluble CD25 (unhelpful criterion)	1 (4)	8 (33)	1 (4)	1 (4)	13 (54)	9 (38)	2 (8)
Fibrinogen (absence of consensus)	0 (0)	13 (54)	9 (38)	2 (8)	0 (0)	13 (54)	11 (46)
Triglycerides (absence of consensus)	2 (8)	13 (54)	6 (25)	3 (13)	0 (0)	15 (63)	9 (38)
Levels of transaminases (absence of consensus)	1 (4)	15 (63)	8 (33)	0 (0)	0 (0)	16 (67)	8 (33)
Percentage of glycosylated ferritin (absence of consensus)	0 (0)	2 (8)	12 (50)	1 (4)	9 (38)	2 (8)	13 (54)

*Letters A, B, C, D, or E are codes corresponding to the following answers: A, absolutely required; B, important; C, of minor interest; D, useless; E, not assess(ed/able) in my routine practice environment.

‡ Choice of answer E was unavailable, absent from the menu list of answers.

## Discussion

Using a consensus formation process that involved experts in the field of HS worldwide, this study is the first attempt for eliciting which features are important to consider for a positive diagnosis of reactive HS in adults. This process elicited a consensus of the panel for 9 helpful and 13 non-helpful criteria, while 4 criteria remained non consensual. There are similarities and differences between the criteria mentioned in the 2004 HLH diagnosis guidelines recommended for the primary form of HS [12] and those issued from the present study focussing on adult cases. Hemophagocytic pictures, high ferritin level, fever, cytopenias (and at a partial level, organomegaly) are shared positive criteria. Interestingly, all the experts consider that cytopenias are required to diagnose HS, but the majority assess unilineage cytopenia as “absolutely required” and bilineage cytopenia as “important”. This indicates that experts may accept HS diagnosis in case of unilineage cytopenia unlike the pediatric HLH 2004 criteria. A known predisposing underlying disease is considered of major importance by the expert panel. This has never been outlined in other proposed set of HS diagnosis criteria. Surprisingly, high LDH were outlined in our study while not retained in the HLH 2004 diagnostic guidelines. High LDH level may be found in many mechanisms of cytopenia (hemolysis, bone marrow necrosis, malignancies' bone marrow involvement) and in many infectious diseases and malignancies in absence of HS. The relevance of high LDH for the diagnosis of HS should certainly be challenged with series data. In addition, whereas natural killer cells cytotoxicity and soluble CD25 are included in the strength criteria of the 2004 HLH diagnosis guidelines, these features were mostly labelled as not assessable in routine practice in the present Delphi study. This result highlights that the daily practice of reactive HS is quite different of the medical care of primary HS in very specialized immunopediatric units. The absence of consensus for high triglyceride and low fibrinogen levels that was observed in our study might be related to a more important value of these criteria in the pediatric setting. Such a result highly suggests the estimation of the robustness of these criteria in a large cohort of adults. Overall, the discrepancies between the Delphi survey presented here and the HLH guidelines reinforce the need to develop diagnostic criteria for reactive HS in adults. The results from the present Delphi survey should also be compared to those issued from a recent international survey on diagnostic criteria for macrophage activation syndrome in systemic juvenile idiopathic arthritis [20]. In the latter survey, pediatric rheumatologists were invited to rank the ten most valuable diagnostic criteria among 28 proposed in a listing questionnaire. As observed when comparing our results with the 2004 HLH diagnosis guidelines, the survey shares substantial similar results with those of the present study. For example, bone marrow hemophagocytosis, hyperferritinemia and falling platelet count were selected by more than 80% of the respondents with a median rank of 9, 7, and 6.5, respectively. Many remaining criteria are more difficult to evaluate since the balance between the ranking of a cited criterion and the percentage of experts selecting this criterion regardless of the corresponding ranking was apparently not considered in the interpretation of the results.

The present study has several limits and the first concerns the participating panel. One might argue that the results issued from the study might have been different with another expert panel. However, based on volunteers selected for their scientific production related to HS in adults, the study involved a substantial final number of participants, 24 experts originating from various countries. In addition, the 39% observed rate of response at the last Delphi round in this study compares with that observed in a previous study (45%) based on the same methodology but on a very different topic [18]. Moreover, such a rate is substantially higher than the median rate of 27% reported for Web-based surveys [21]. Considering the size of the panel in our study, the threshold proportion value that was arbitrarily *a priori* chosen for categorizing a given criterion as associated with a positive or a negative consensus (a proportion of 0.75 positive answers or a proportion of 0.75 negative answers, respectively) corresponds to a 95% confidence range of [0.548; 0.883] [22], above 50%. This highly suggests that consensus items issued from the study are rather solid and are likely to reflect what would be obtained with a larger or different panel. The remaining 4 criteria i.e., those for which a consensus was not reached deserve some comments: the three criteria (fibrinogen, triglycerides, levels of transaminases) which roughly received one half to two-thirds of positive judgments should be further investigated in future studies in order to get more insight on their true value. In contrast, the percentage of glycosylated ferritin does not merit further investigations: a substantial part of the experts considered that it was not assessable in routine practice (38%) and most experts (54%) negatively categorized this criterion. Another issue concerns the selection of criteria chosen for composing the survey but such an issue appears less questionable: we deliberately chose to focus experts' attention to all features mentioned in the initial bibliographic search, to avoid our *a priori* opinions on the relevance of any criterion. The study did not investigate the threshold value for quantitative criteria (e.g., the threshold value that should be used for considering a ferritin level as “high”), and at first sight, one might consider that this is a third limit of the study. However, Delphi studies are poorly adapted to such investigations because many rounds may be required for eliciting a consensual threshold value while a disaffection of experts is likely to occur with time.

In this study, we chose to elicit criteria of interest: the present study should be viewed as the first step for defining international guidelines devoted to the positive diagnosis of reactive HS. In a second step, these criteria should be compared against historical cases to evaluate their utility. A study contrasting the 9 criteria for which there was a positive consensus and 3 of the 4 criteria for which there was an absence of consensus with case and control adults has been conducted, and the publication of the results is pending.

## Supporting Information

Figure S1 **Preview screen explaining or repeating (displayed at each connexion to survey) to experts how to complete the questionnaire.**
(PDF)

Figure S2 **A typical screen displayed during the completion of the questionnaire in the second Delphi round.**
(PDF)

Figure S3 **Distribution of the answers at the first and second delphi round, for each of the 26 questionnaire items.**
(PDF)

Table S1 **Complete survey data: answers of each participant to the questionnaire at each Delphi round.** ^*^Each of the 26 participants is arbitrarily labelled with a number. **^†^**Each string composed of 26 letters is a coded pattern representing the whole set of answers of a given expert at a given Delphi round (26 answers): the 26 question wordings are shown in Table 1 of the main manuscript whereas letters A, B, C, D, or E in the string are codes corresponding to the following answers: A, absolutely required; B, important; C, of minor interest; D, useless; E, not assessable in my routine practice environment. ^‡^Experts #5 and #10 did not participate in the second round.
(PDF)
