# Supplementary material for: A Web-Based Delphi Study for Eliciting Helpful Criteria in the Positive Diagnosis of Hemophagocytic Syndrome in Adult Patients
Source: PLoS One. 2014 Apr 7;9(4):e94024. doi: 10.1371/journal.pone.0094024 (PMC3977971; doi:10.1371/journal.pone.0094024)
Supplement: Figure S1 — Preview screen explaining or repeating (displayed at each connexion to survey) to experts how to complete the questionnaire. (PDF) [file pone.0094024.s001.pdf]

The questionnaire is composed of 26 questions.

Each question addresses the helpfulness of a criterion in the diagnosis of a reactive hemophagocytic syndrome in adults. In order to facilitate the completion of the survey, questions were all designed with the same pattern. You will be asked to answer the question by choosing one of the 5 proposed answers

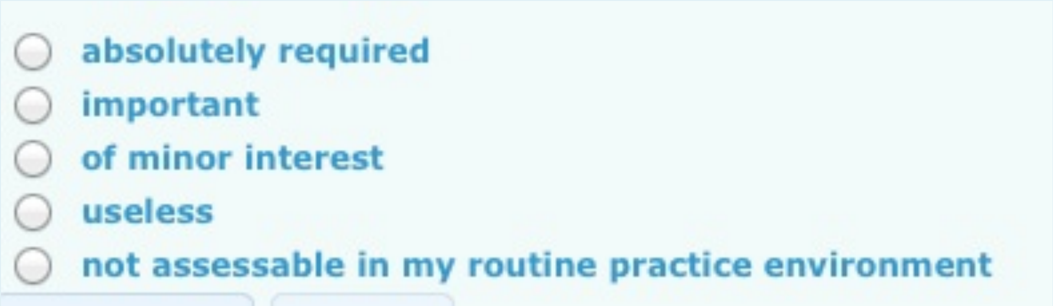

(n.b. the fifth proposition, “not assessable …”, is not proposed in questions 1 to 5) defined as follows:

absolutely required: the absence of the criterion would make the diagnosis of hemophagocytic syndrome very unlikely.

important: the absence of the criterion would not exclude the possibility of hemophagocytic syndrome but its presence clearly strengthens the diagnosis.

of minor interest: the presence of the criterion may help you in diagnosing hemophagocytic syndrome, but the absence of the criterion does not influence much your diagnosis.

useless: you do not mind the presence or absence of the criterion for diagnosing hemophagocytic syndrome

not assessable in my routine practice environment: Whatever the potential helpfulness of the criterion in the diagnosis, it is never assessed in your clinical practice, either because other criteria are sufficient for the diagnosis of hemophagocytic syndrome or because it is not technically possible to assess this criterion in your department/hospital.
