# Supplementary material for: A Web-Based Delphi Study for Eliciting Helpful Criteria in the Positive Diagnosis of Hemophagocytic Syndrome in Adult Patients
Source: PLoS One. 2014 Apr 7;9(4):e94024. doi: 10.1371/journal.pone.0094024 (PMC3977971; doi:10.1371/journal.pone.0094024)
Supplement: Figure S2 — A typical screen displayed during the completion of the questionnaire in the second Delphi round. (PDF) [file pone.0094024.s002.pdf]

MASCrit_DelphiSurvey survey, round 2 , *16 questions left*

Question 11:

For the positive diagnosis of reactive hemophagocytic syndrome, high levels of transaminases (i.e. SGOT, SGPT) are:

- ○ absolutely required
- ○ important
- ○ of minor interest
- ○ useless
- ○ not assessable in my routine practice environment

✔ Validate ▸▸ Skip

Number of answers at the previous iteration: 26

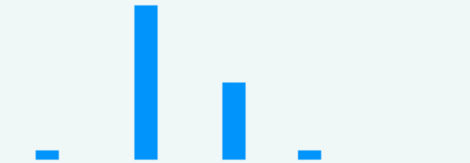

absolutely required	important	of minor interest	useless	not assessable in my routine practice environment
1	16	8	1	0
	YOU			
