# Supplementary material for: A Web-Based Delphi Study for Eliciting Helpful Criteria in the Positive Diagnosis of Hemophagocytic Syndrome in Adult Patients
Source: PLoS One. 2014 Apr 7;9(4):e94024. doi: 10.1371/journal.pone.0094024 (PMC3977971; doi:10.1371/journal.pone.0094024)

Q1: Predisposing underlying disease.

For the positive diagnosis of reactive hemophagocytic syndrome, a predisposing underlying disease (e.g., lupus erythematosus, HIV, Still disease, lymphoma, drug-induced immunodepression) is:

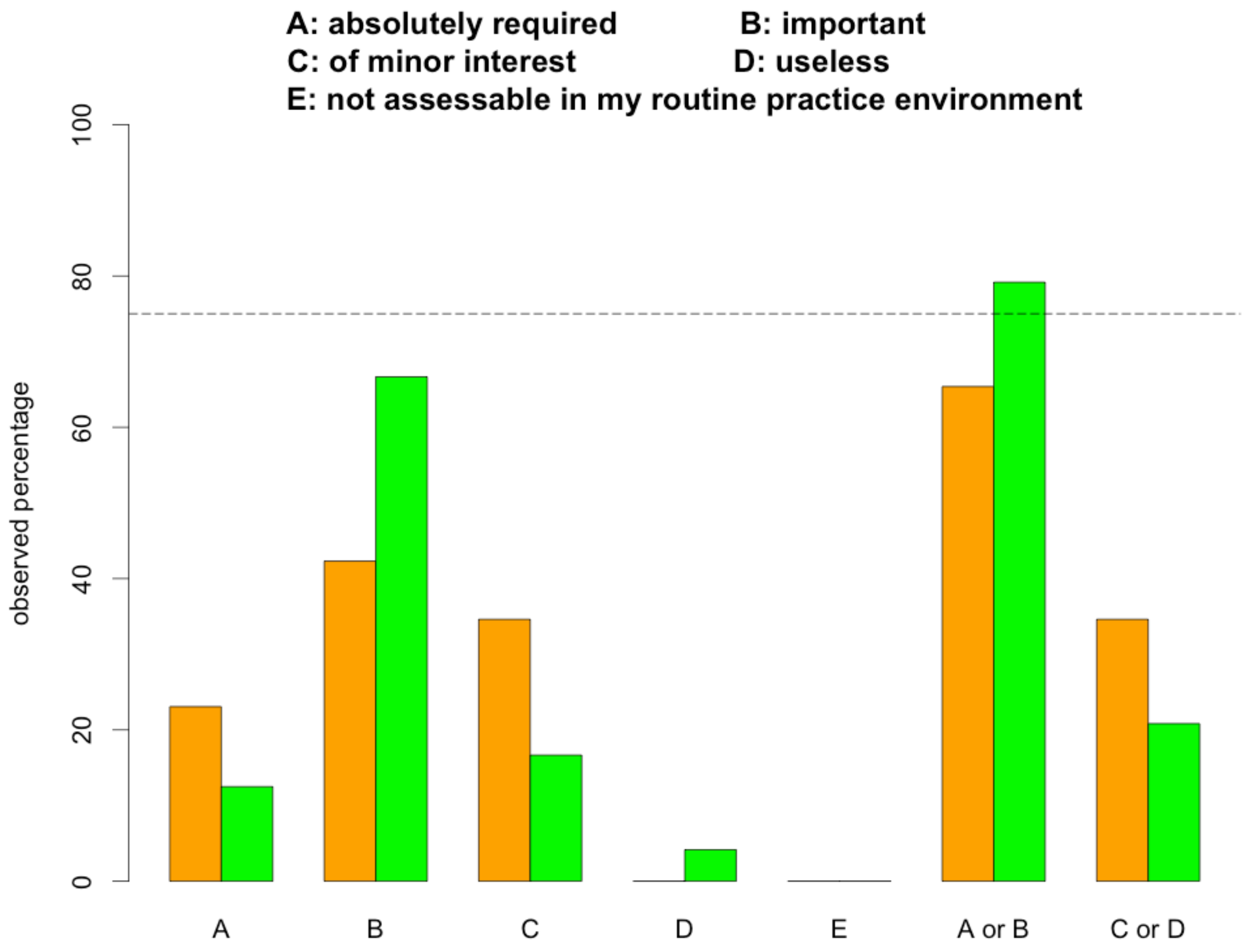

Q2: Fever.
For the positive diagnosis of reactive hemophagocytic syndrome, fever is:

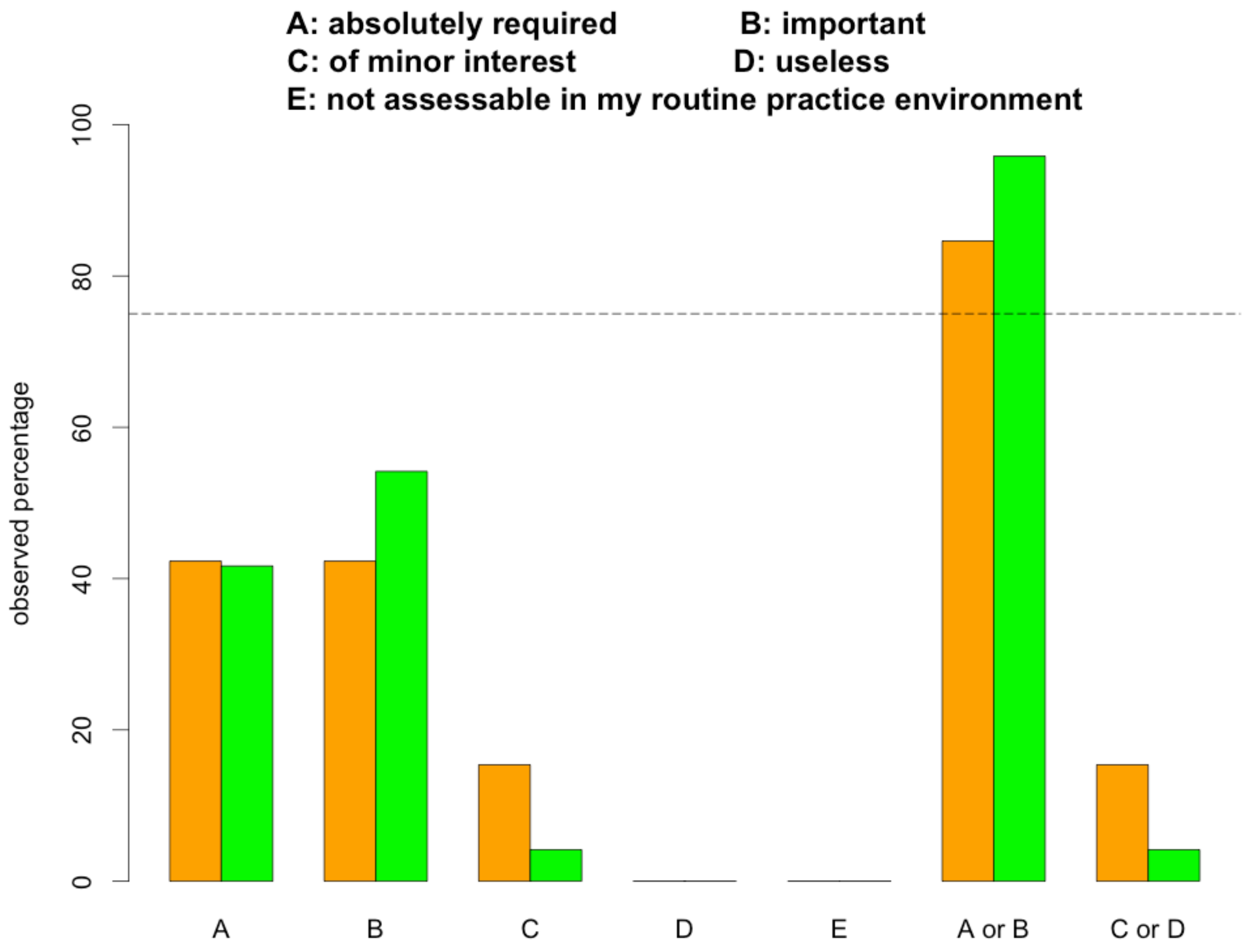

Q3. Organ failure.
For the positive diagnosis of reactive hemophagocytic syndrome, the presence of an organ failure (e.g. renal insufficiency, cardiac failure, respiratory distress syndrome) is:

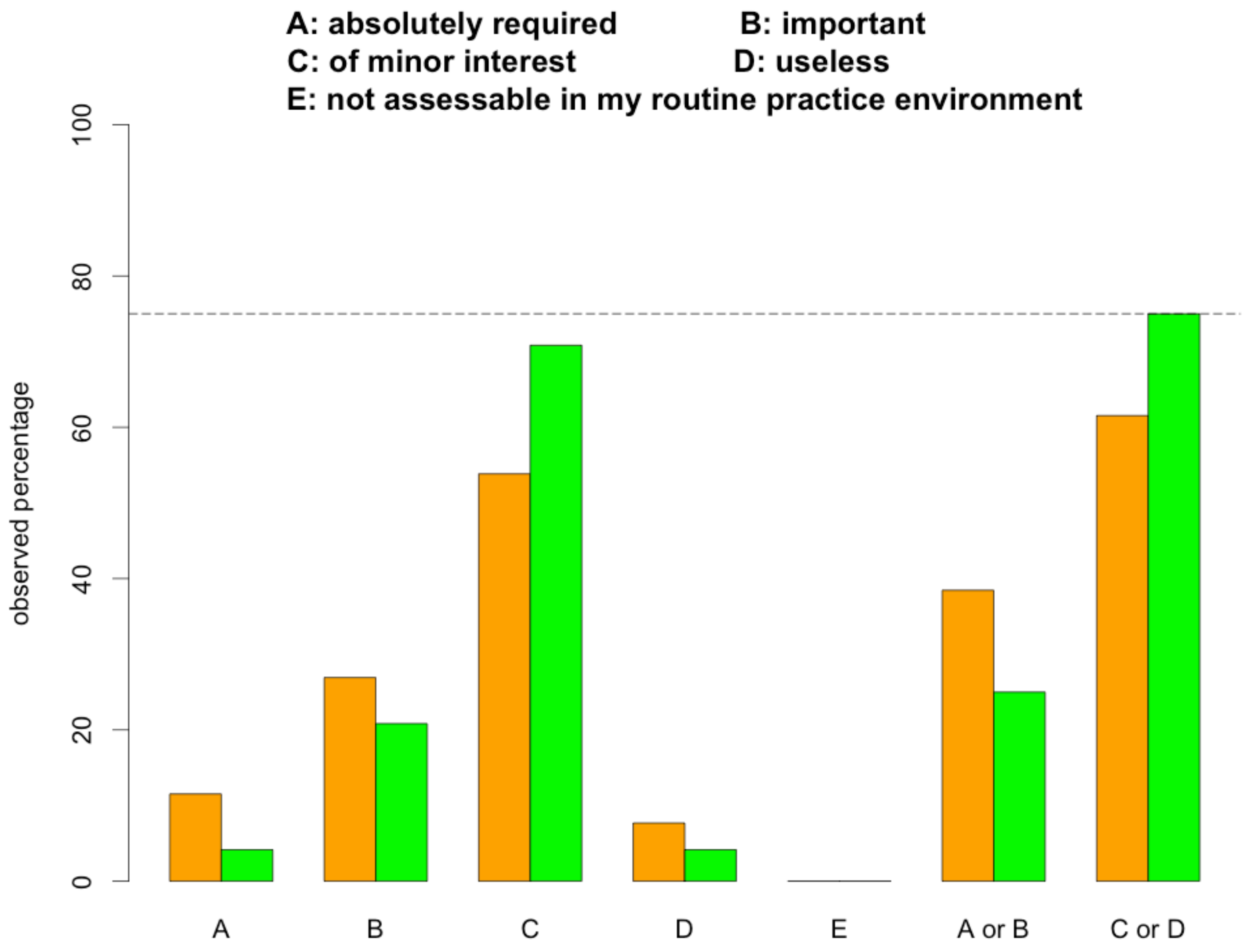

Q4: Organomegaly.

For the positive diagnosis of reactive hemophagocytic syndrome, organomegaly (i.e., hepatomegaly, splenomegaly and/or adenomegaly) is:

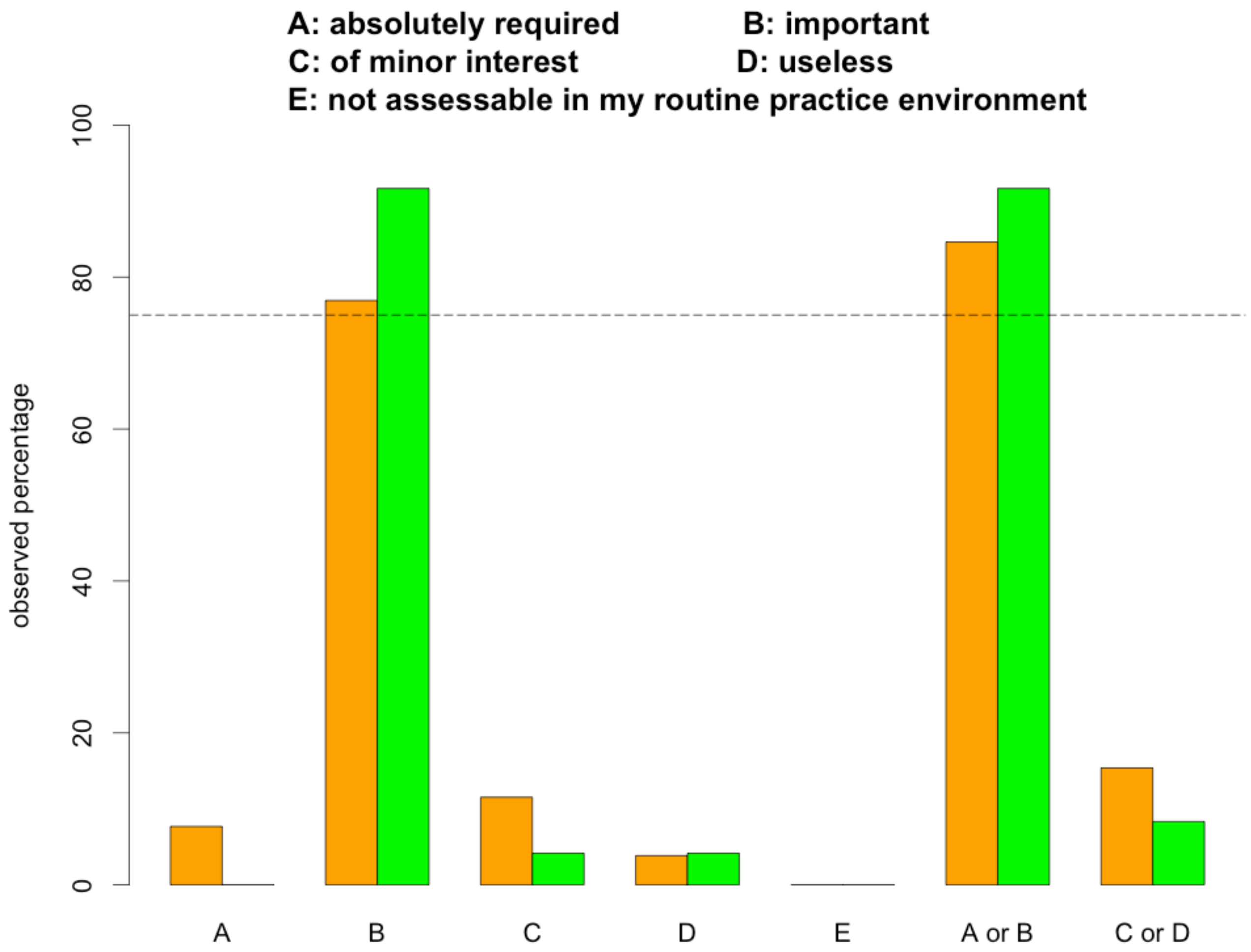

Q5: Maculo-papular cutaneous rash.

For the positive diagnosis of reactive hemophagocytic syndrome, the presence of a maculo-papular cutaneous rash is:

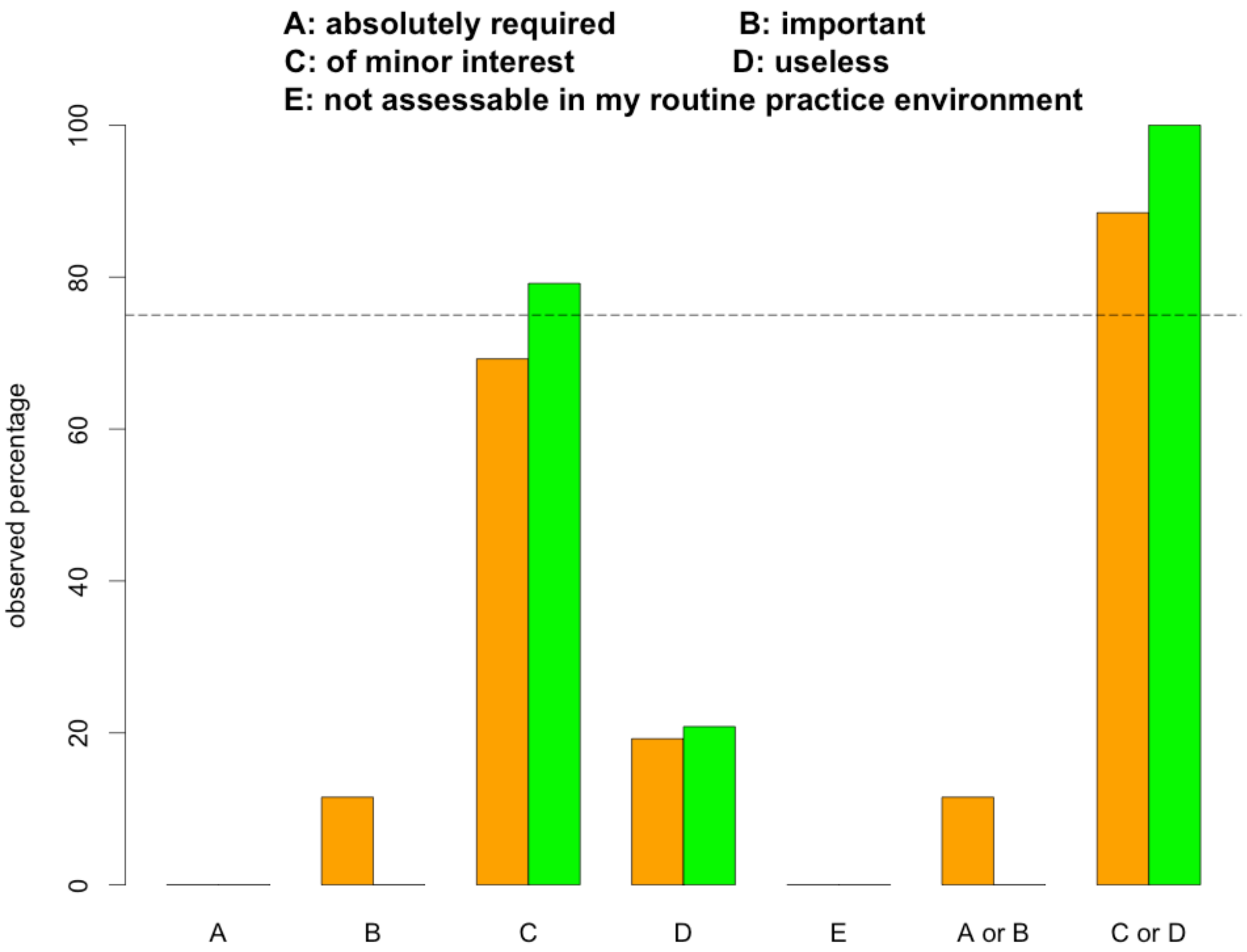

Q6: Unilineage cytopenia.

For the positive diagnosis of reactive hemophagocytic syndrome, unilineage cytopenia (i.e. anemia or thrombocytopenia or leucopenia) is:

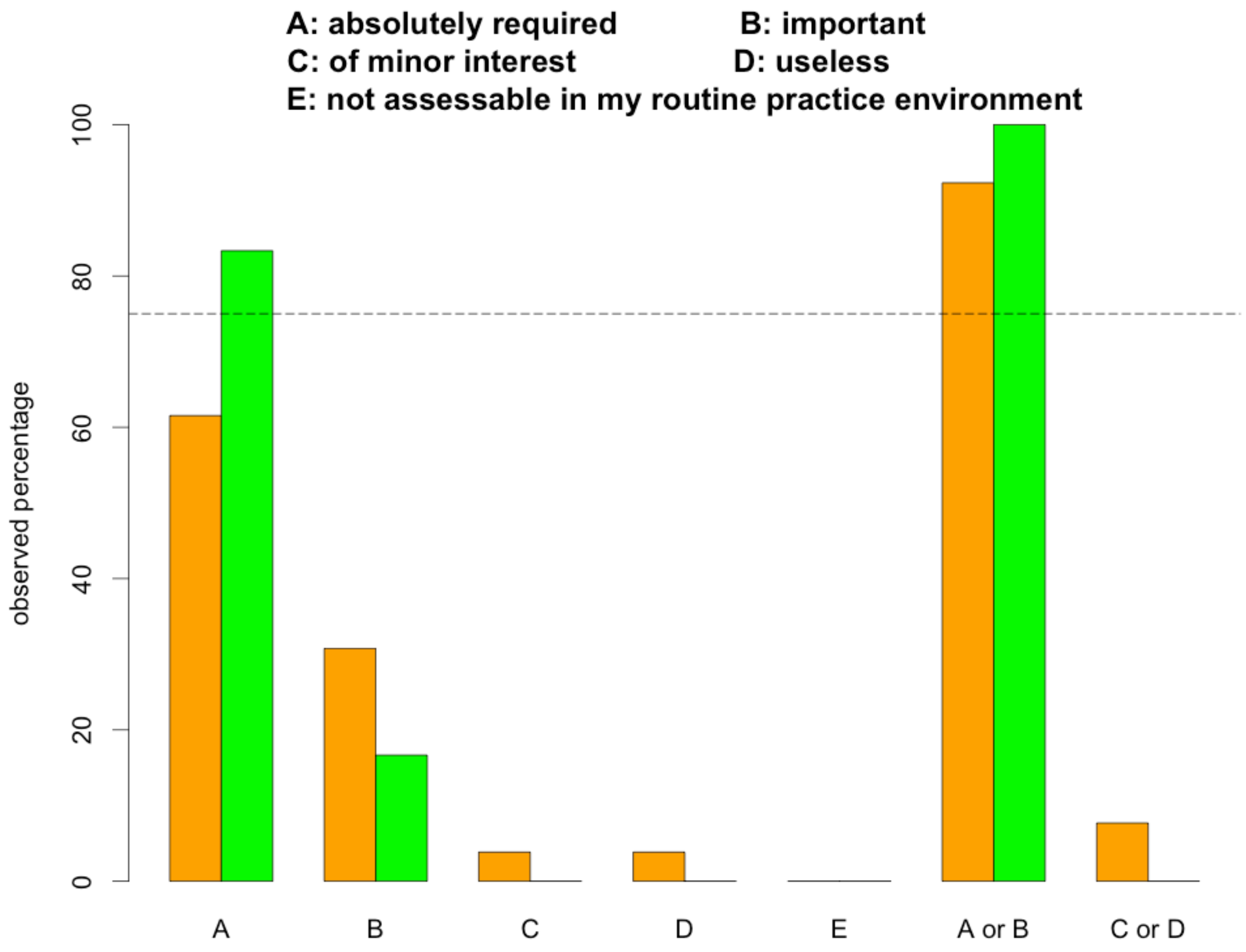

Q7: Bicytopenia.

For the positive diagnosis of reactive hemophagocytic syndrome, bicytopenia (e.g. anemia + thrombocytopenia, leucopenia + thrombocytopenia, anemia + leucopenia) is:

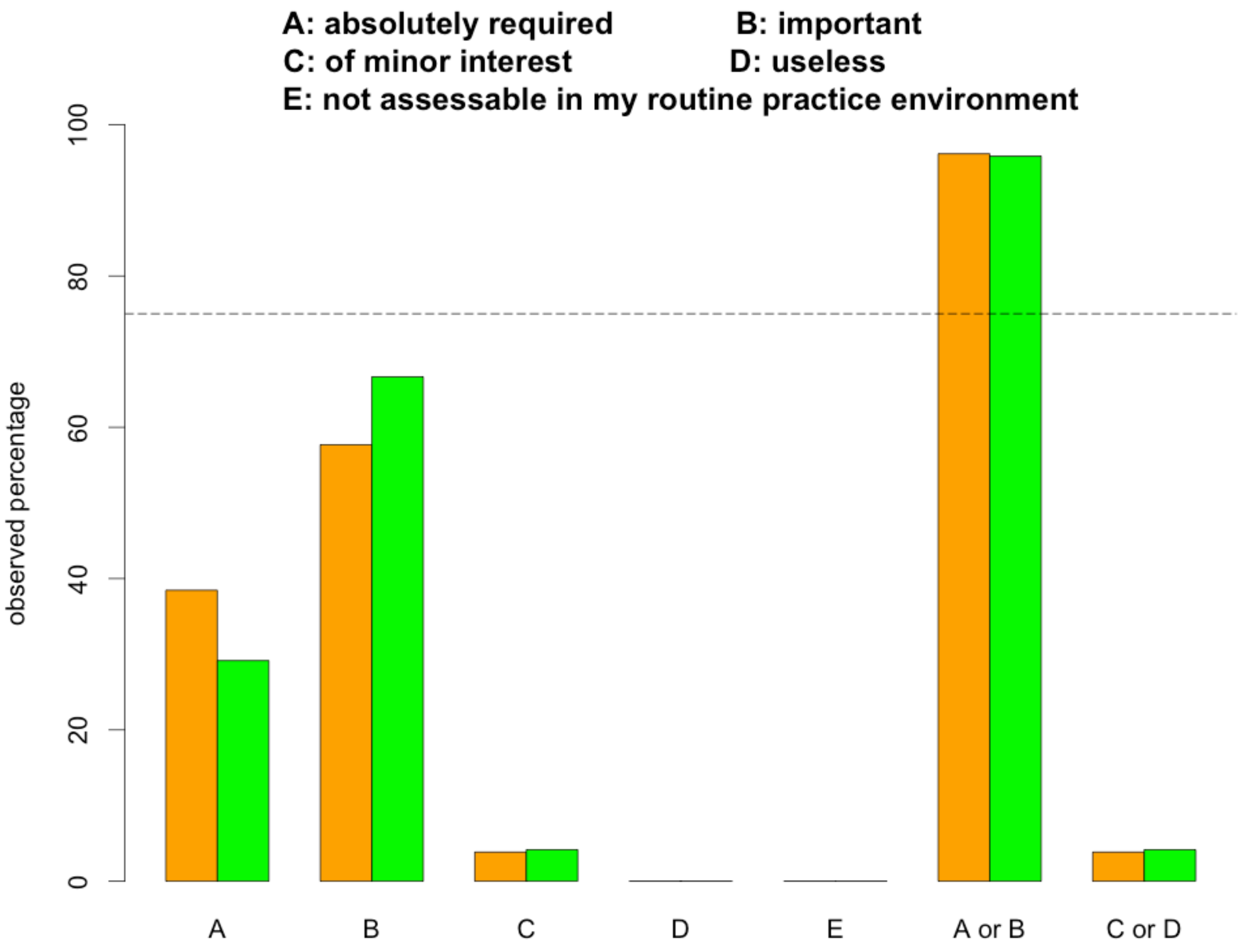

Q8: Pancytopenia.

For the positive diagnosis of reactive hemophagocytic syndrome, pancytopenia is:

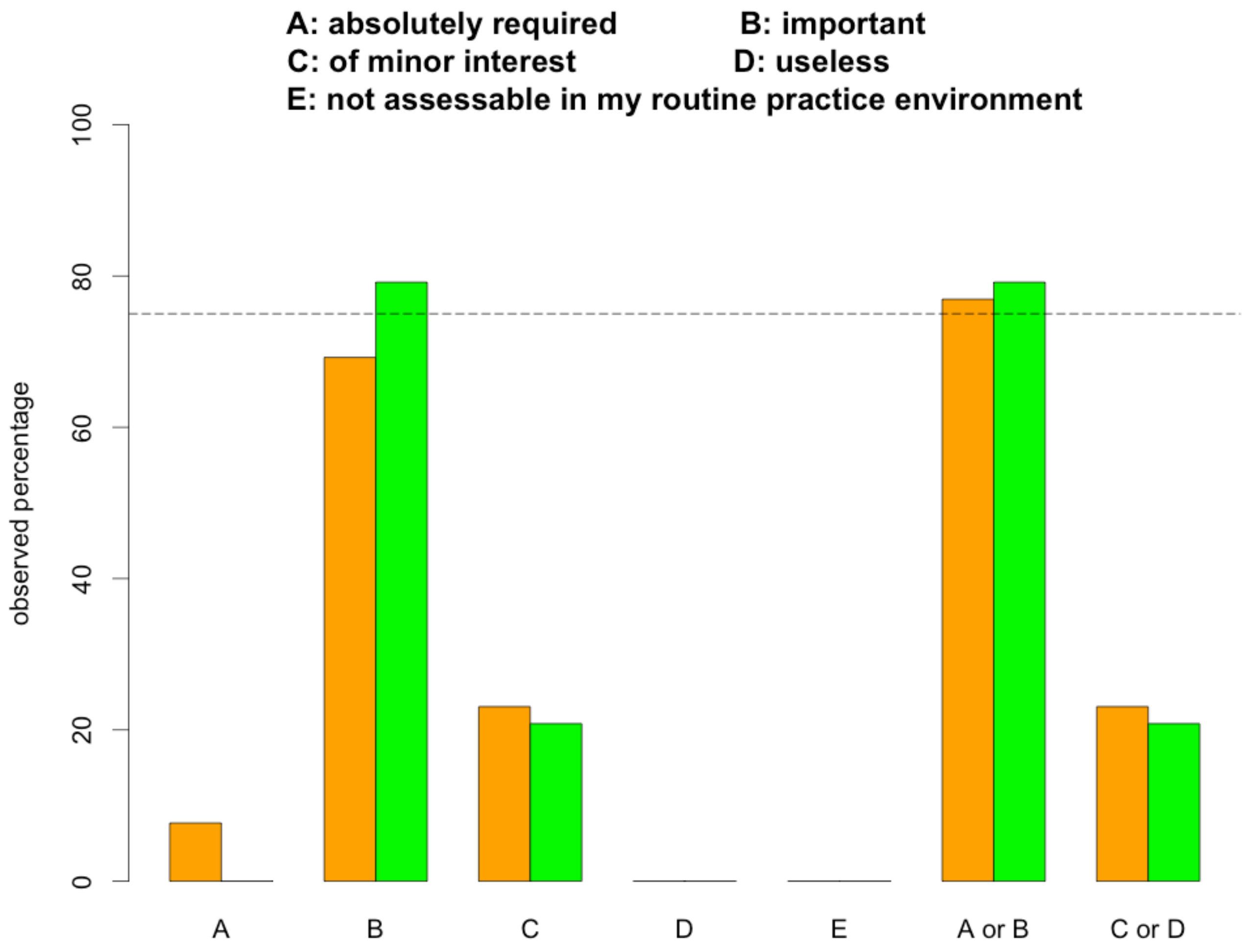

Q9: Ferritin level.
For the positive diagnosis of reactive hemophagocytic syndrome, a high ferritin level is:

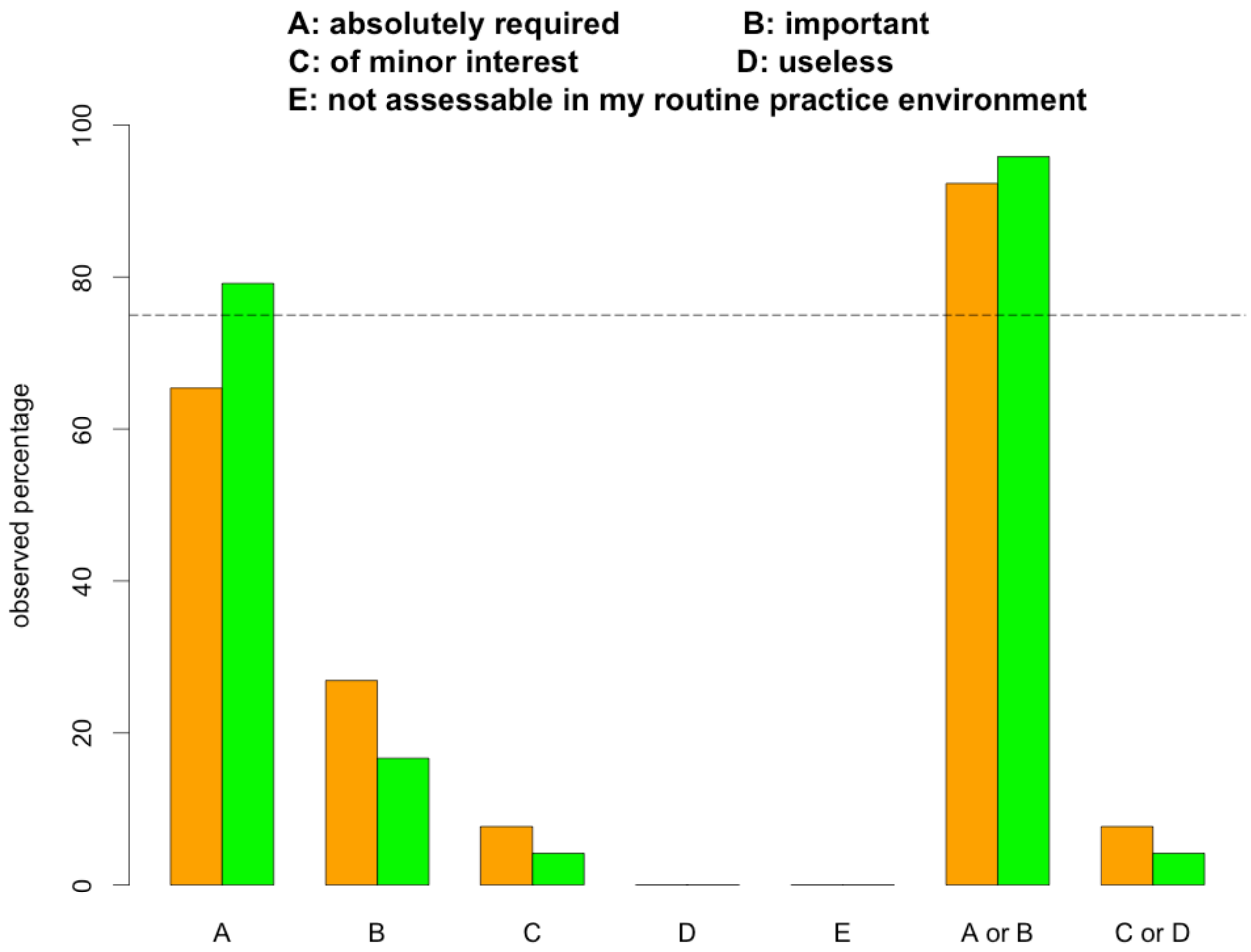

Q10: Percentage of glycosylated ferritin.

For the positive diagnosis of reactive hemophagocytic syndrome, a low percentage of glycosylated ferritin is:

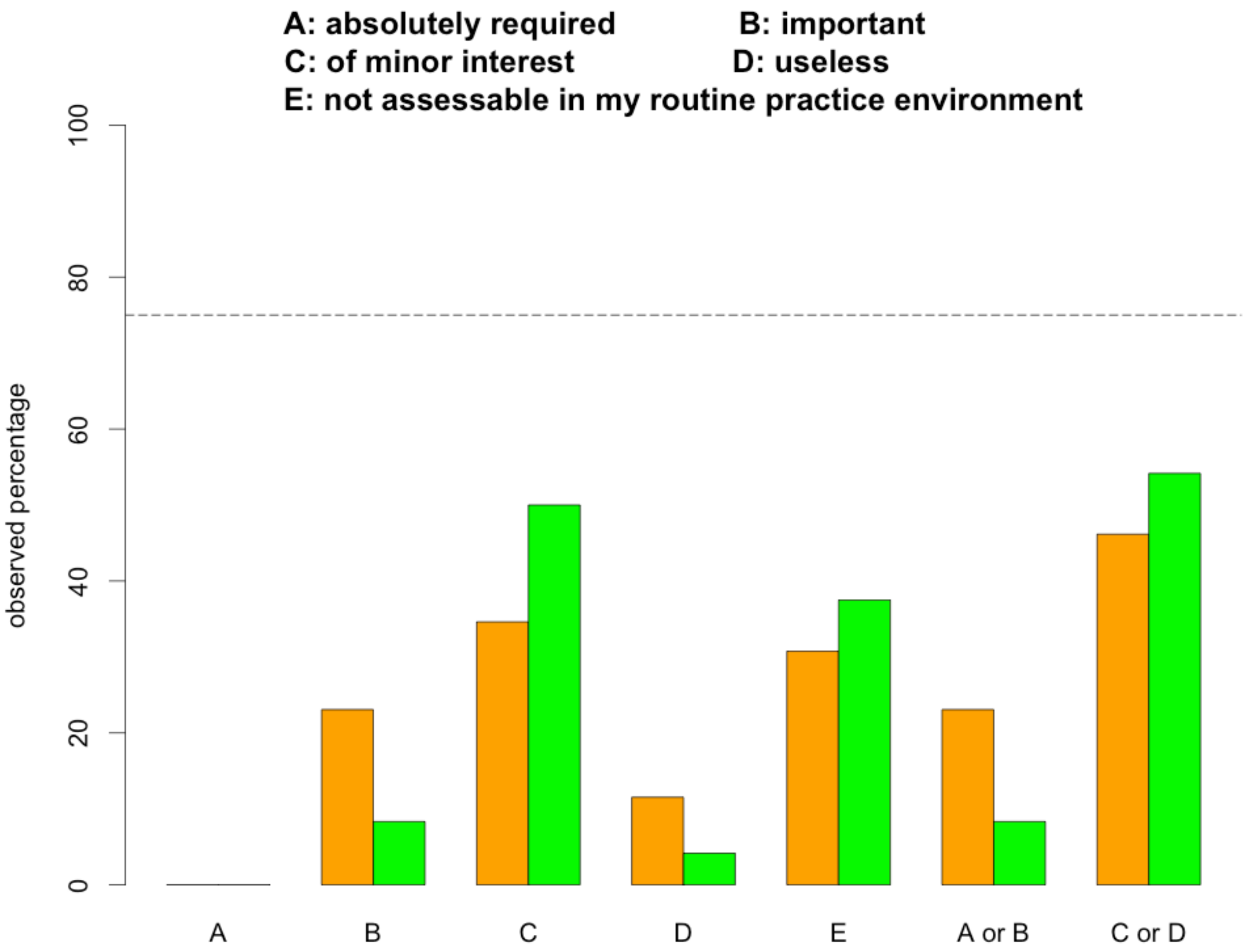

Q11: Levels of transaminases.

For the positive diagnosis of reactive hemophagocytic syndrome, high levels of transaminases (i.e. SGOT, SGPT) are:

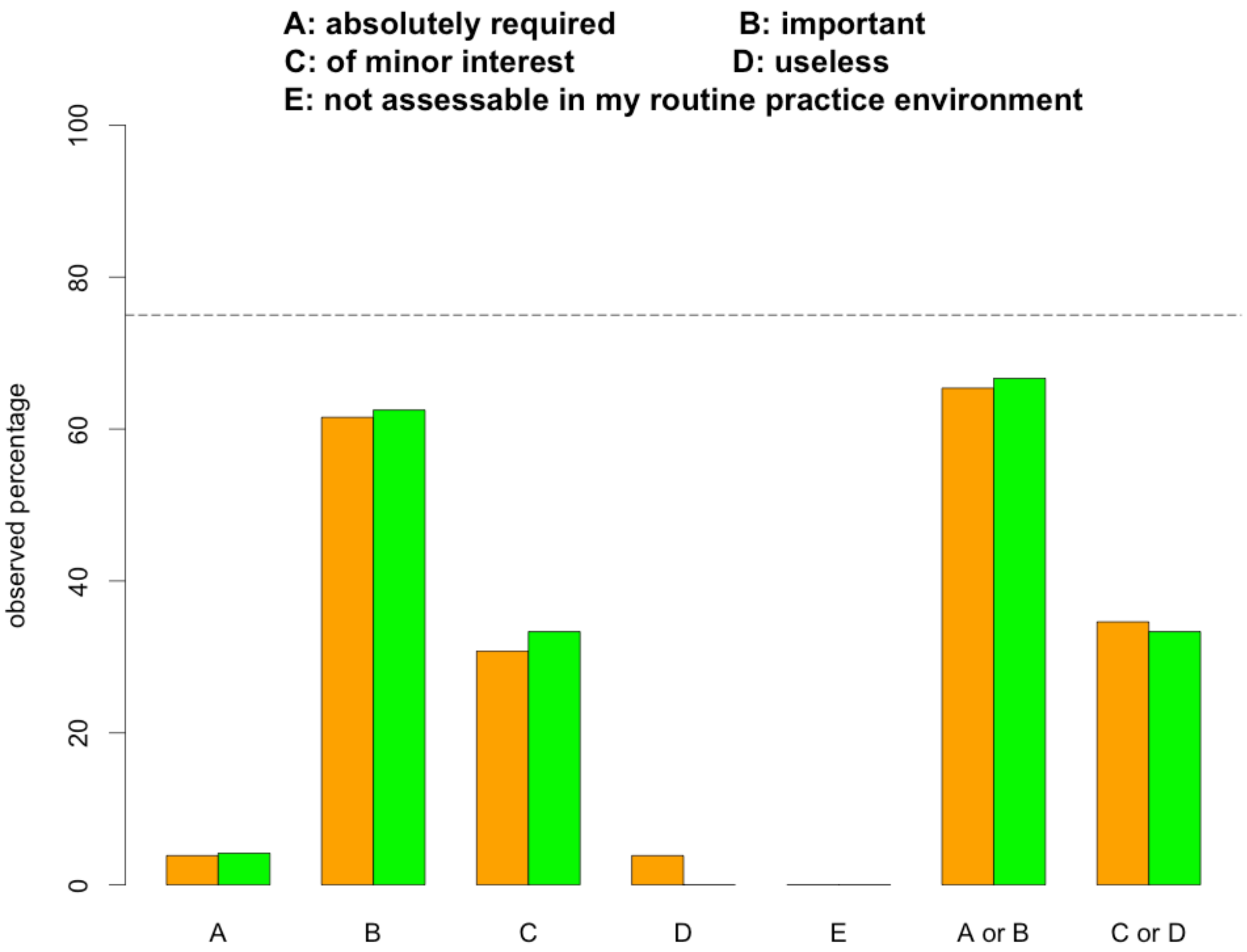

Q12: Bilirubin.
For the positive diagnosis of reactive hemophagocytic syndrome, a high level of total bilirubin is:

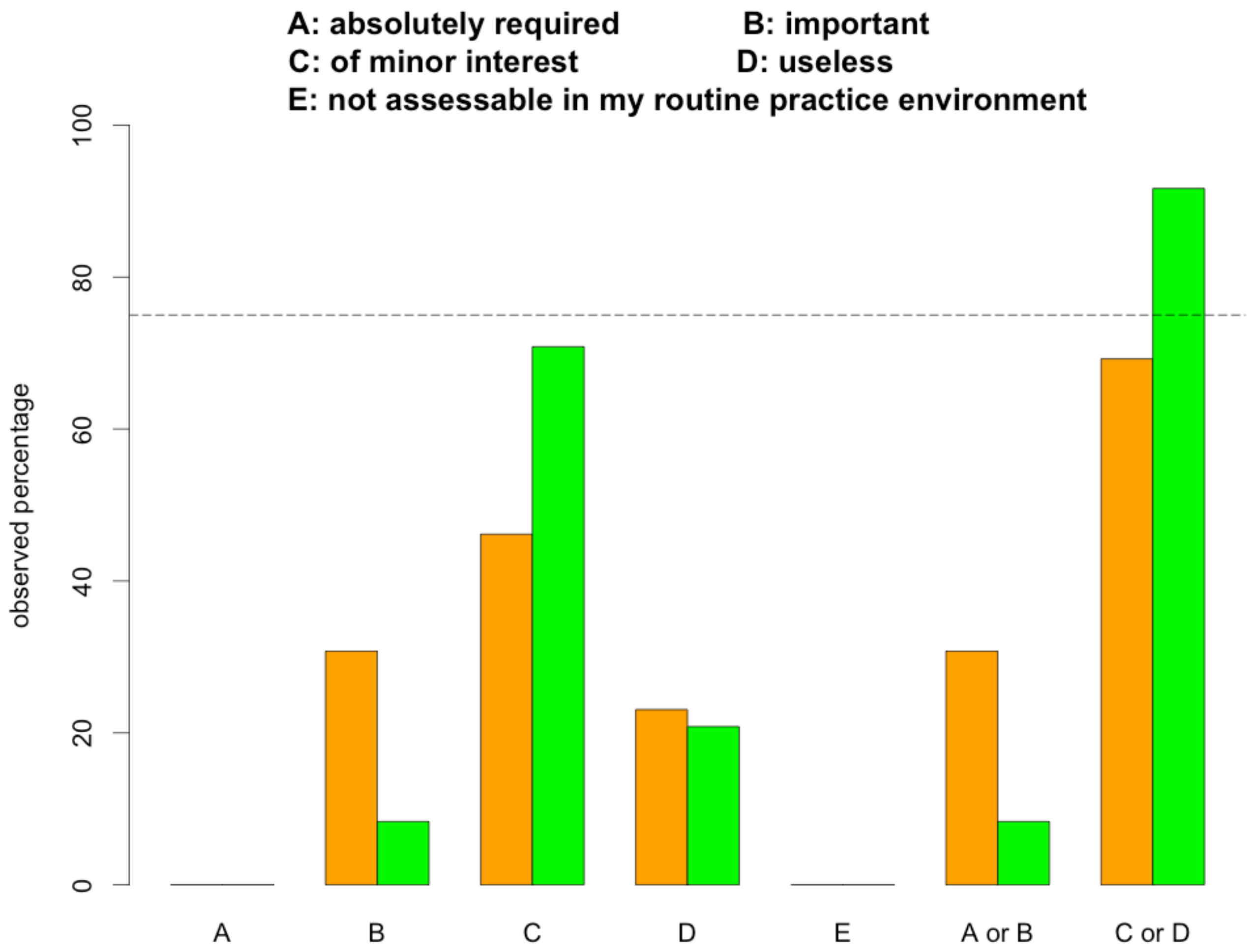

Q13: Gamma glutamyl transferase.
For the positive diagnosis of reactive hemophagocytic syndrome, a high level of gamma glutamyl transferase is:

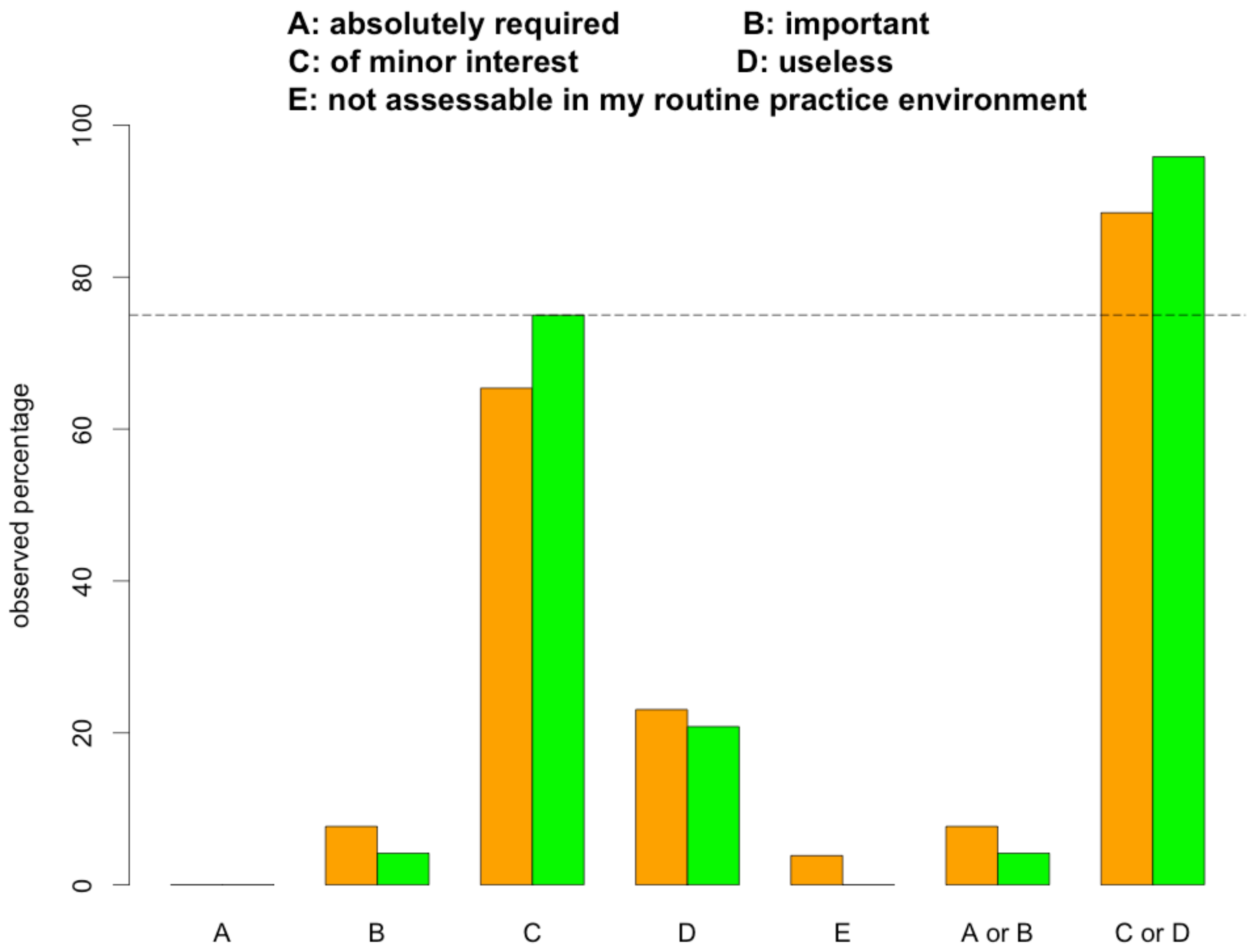

Q14: Hyponatremia.
For the positive diagnosis of reactive hemophagocytic syndrome, hyponatremia is:

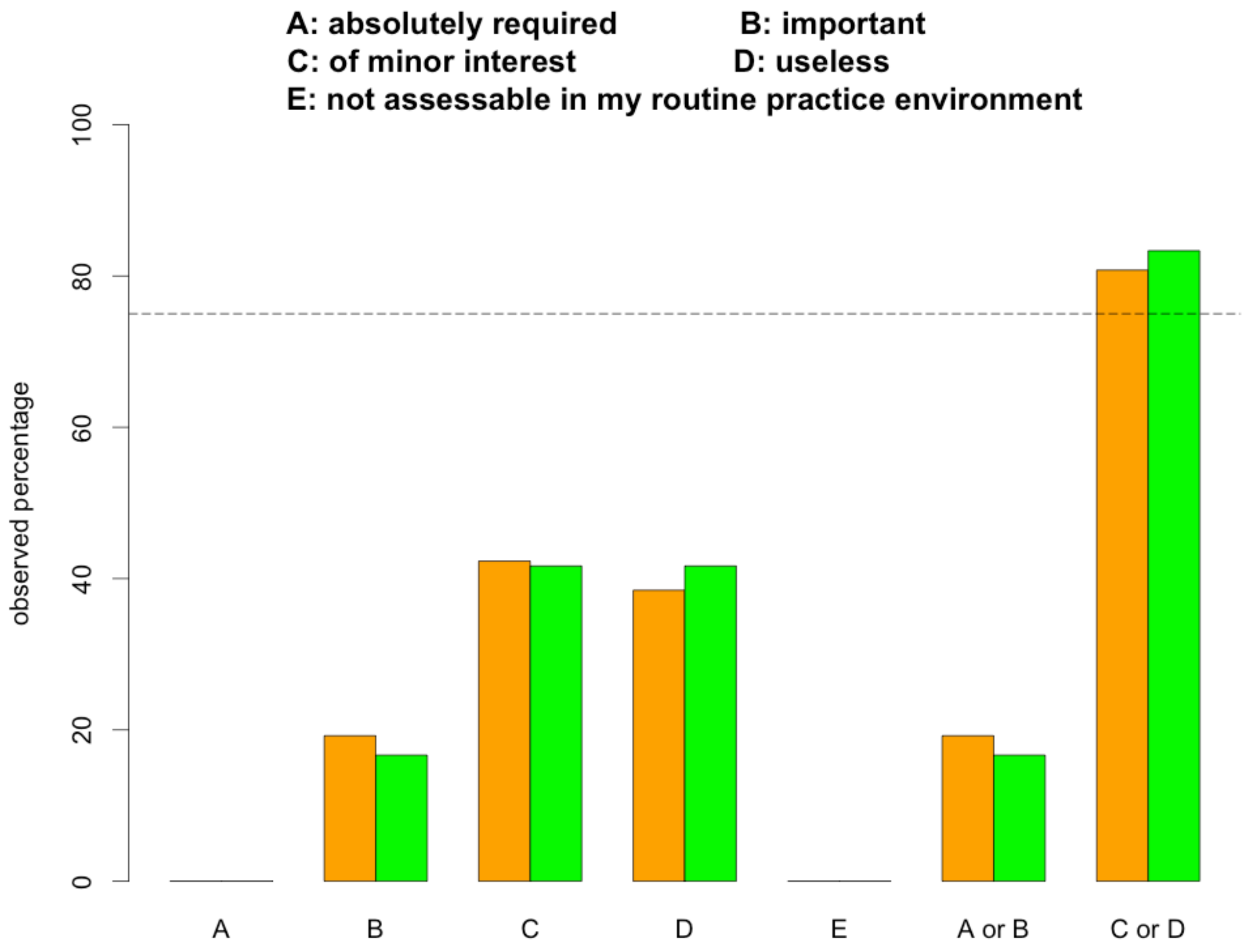

Q15: Fibrinogen level.
For the positive diagnosis of reactive hemophagocytic syndrome, a low fibrinogen level is:

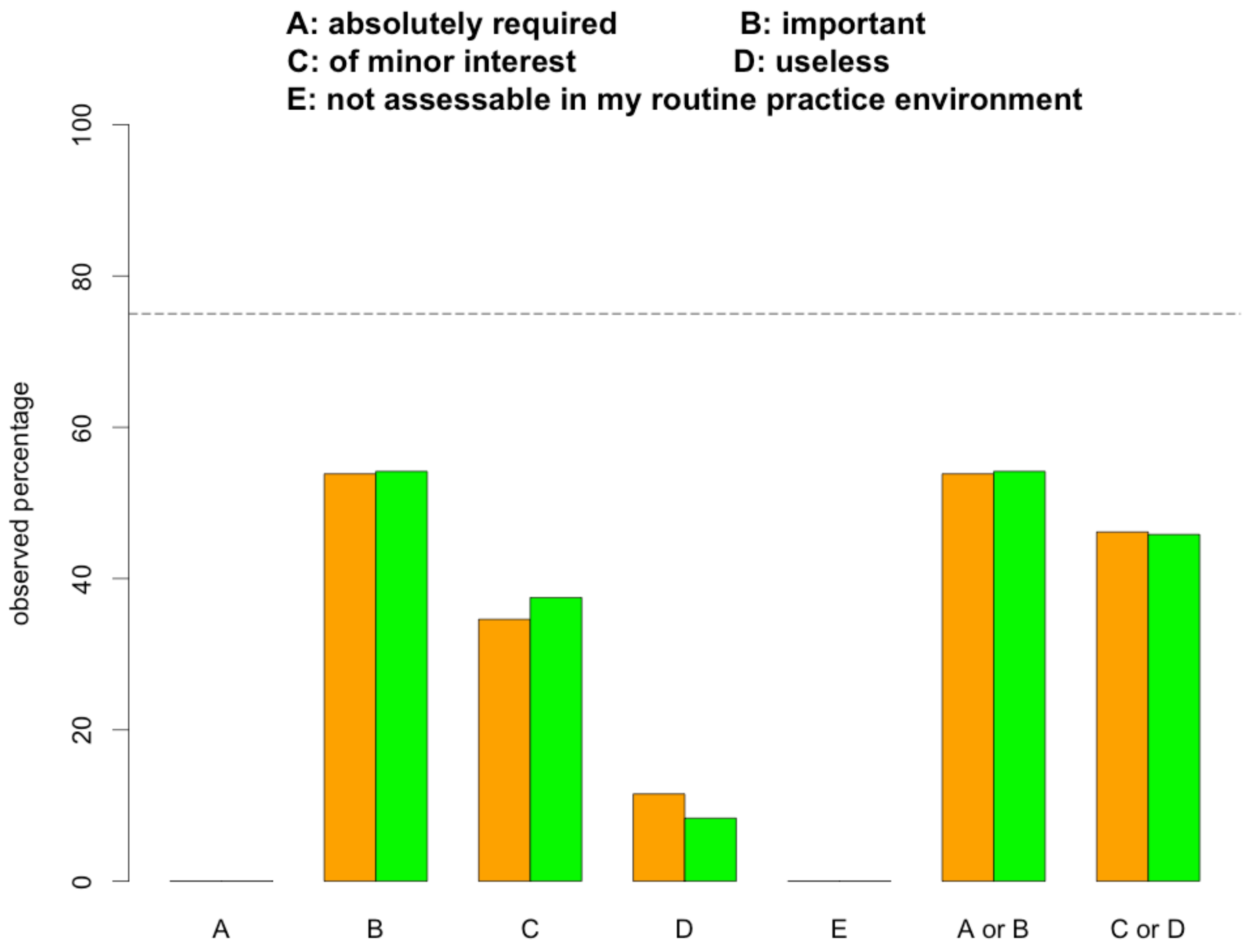

Q16: Triglyceride level.
For the positive diagnosis of reactive hemophagocytic syndrome, a high triglyceride level is:

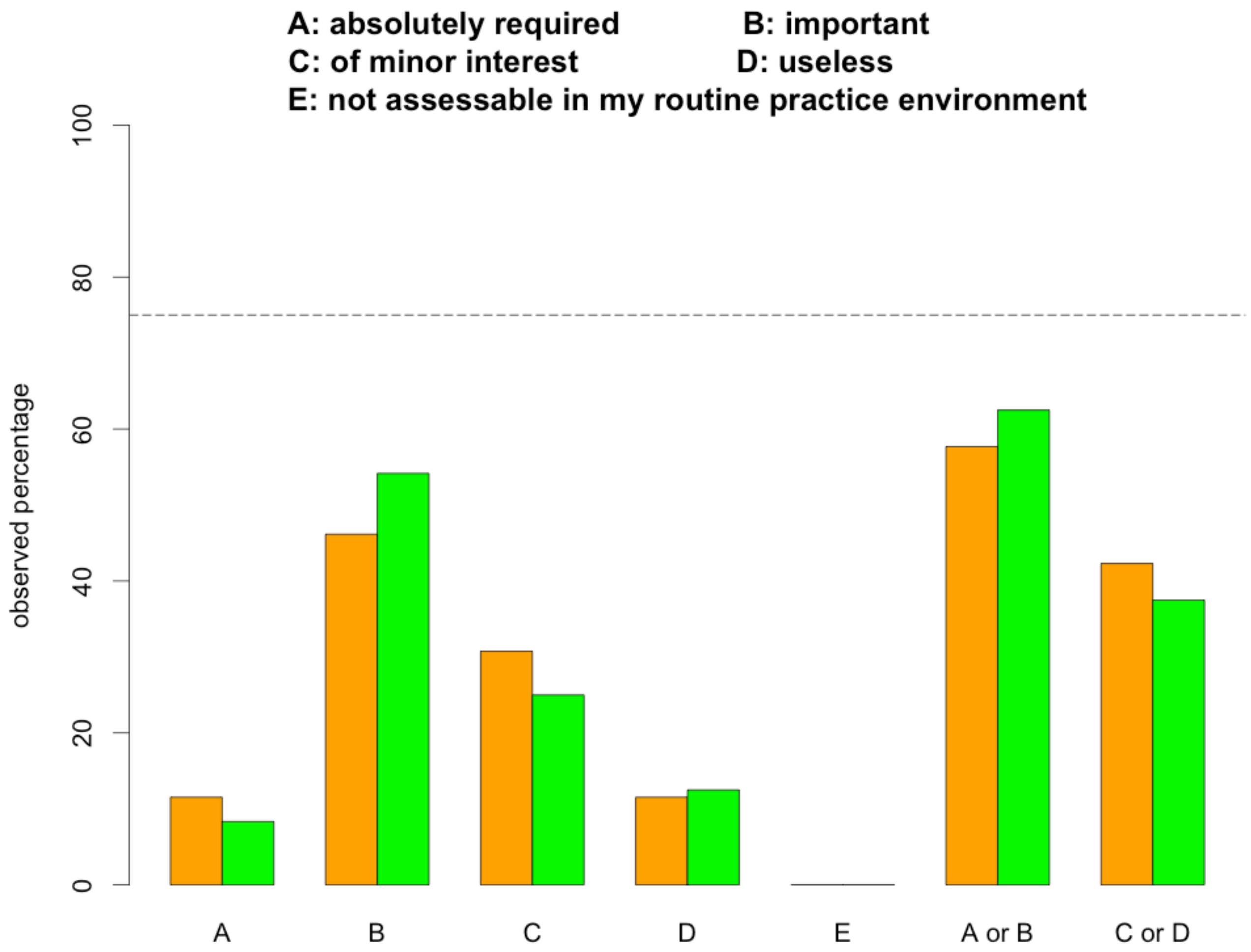

Q17: Lactate dehydrogenase.

For the positive diagnosis of reactive hemophagocytic syndrome, a high level of lactate dehydrogenase is:

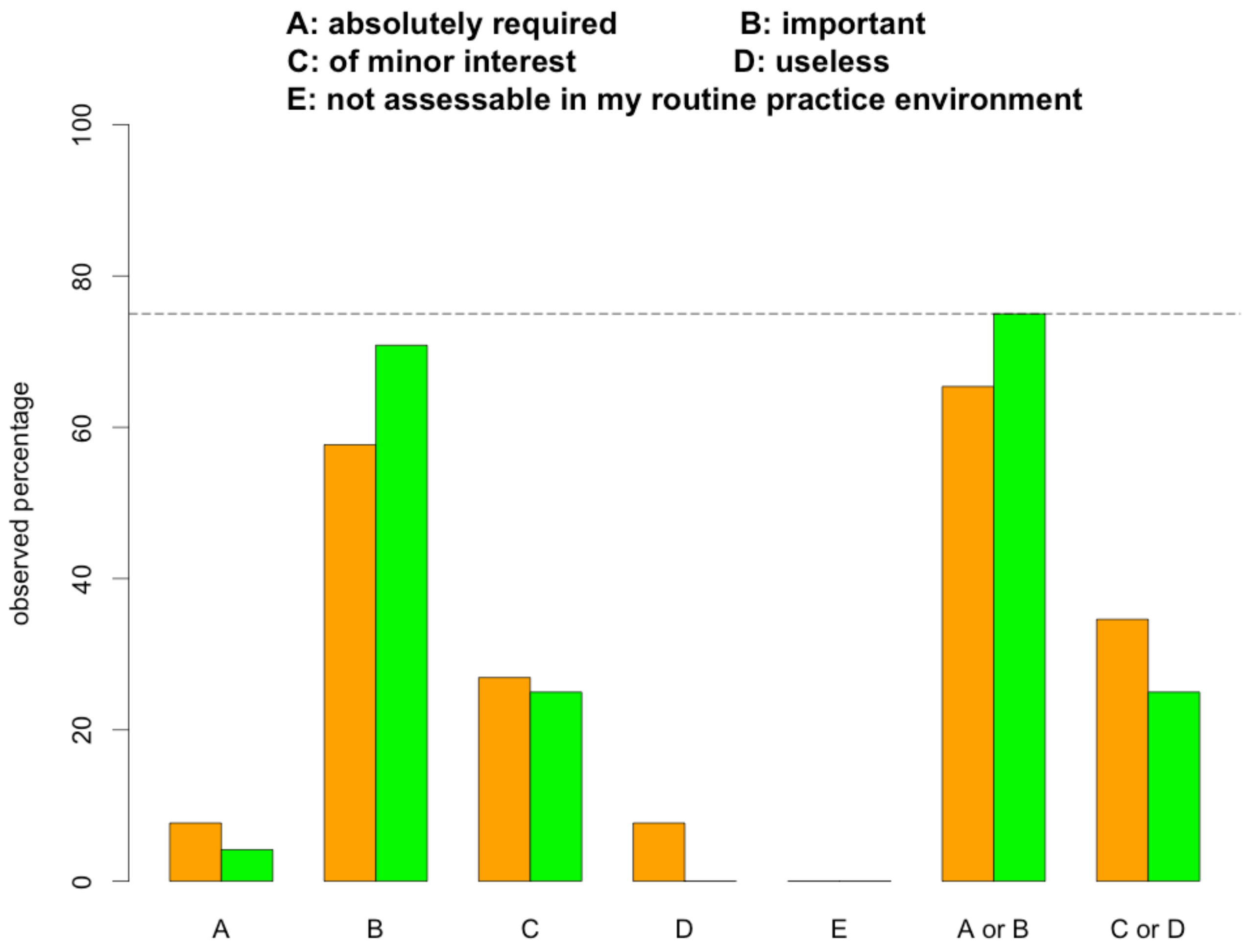

Q18: Serum interleukin-2.

For the positive diagnosis of reactive hemophagocytic syndrome, a high level of serum interleukin-2 is:

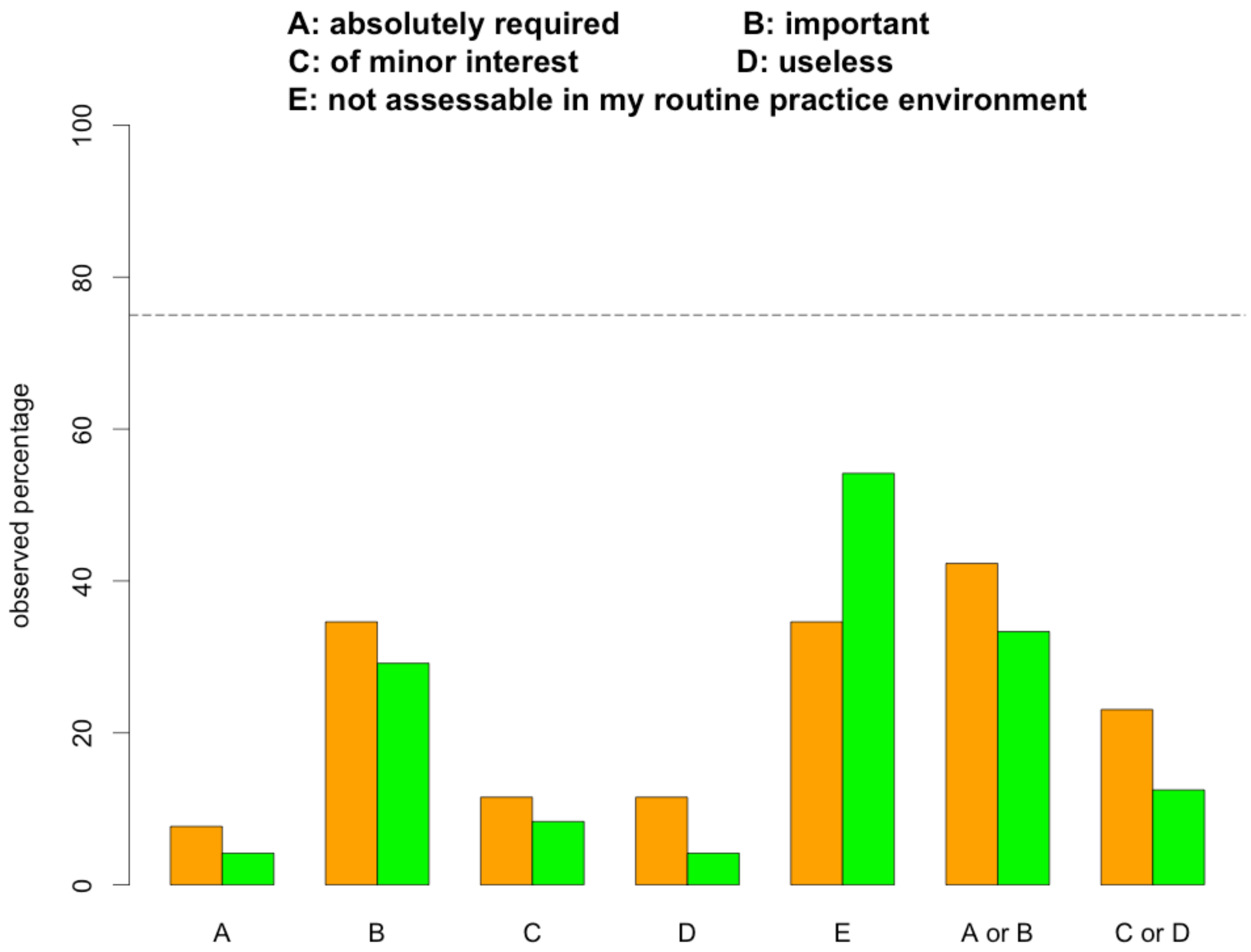

Q19: Soluble CD163.
For the positive diagnosis of reactive hemophagocytic syndrome, a high level of soluble CD163 is:

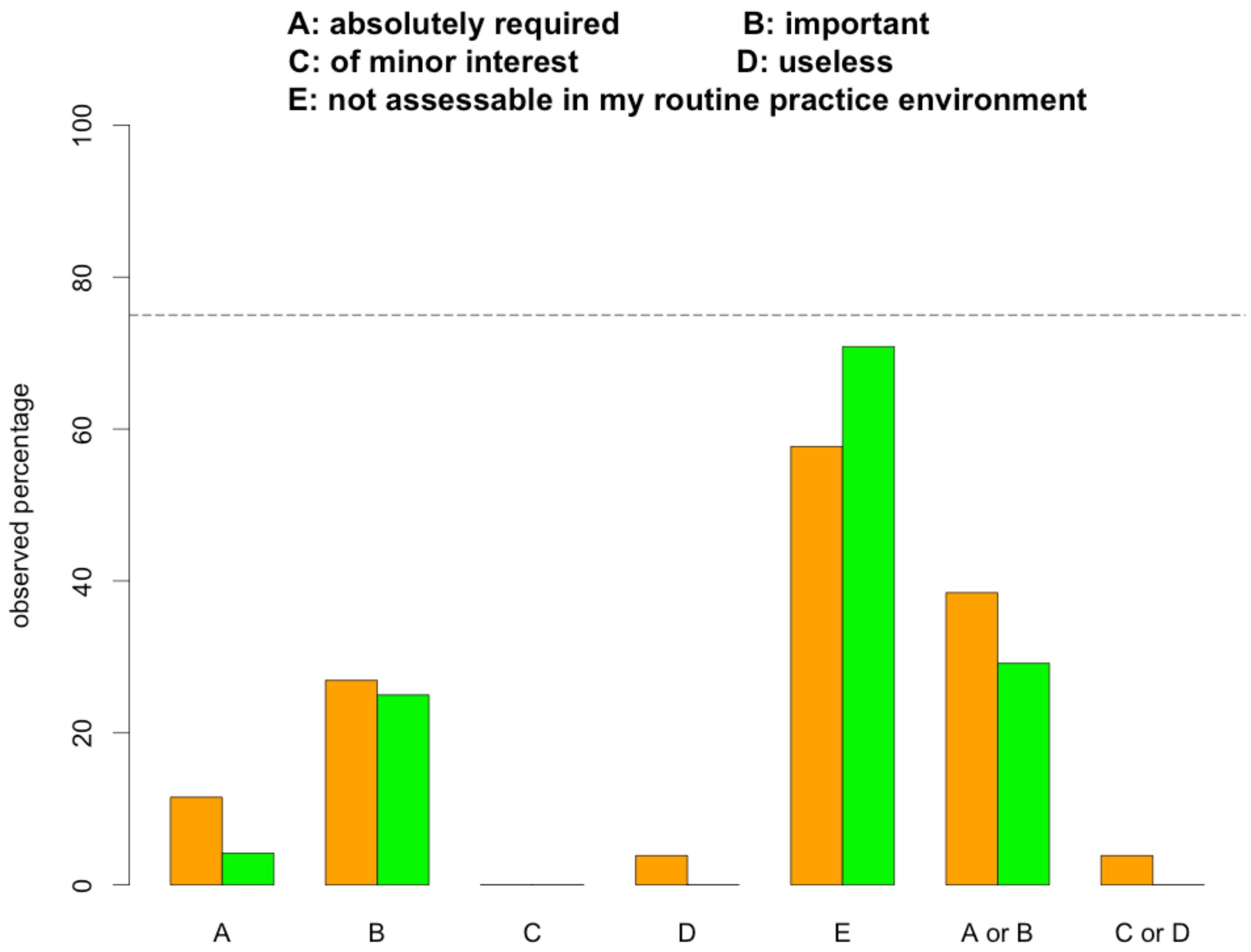

Q20: Soluble CD25.
For the positive diagnosis of reactive hemophagocytic syndrome, a high level of soluble CD25 is:

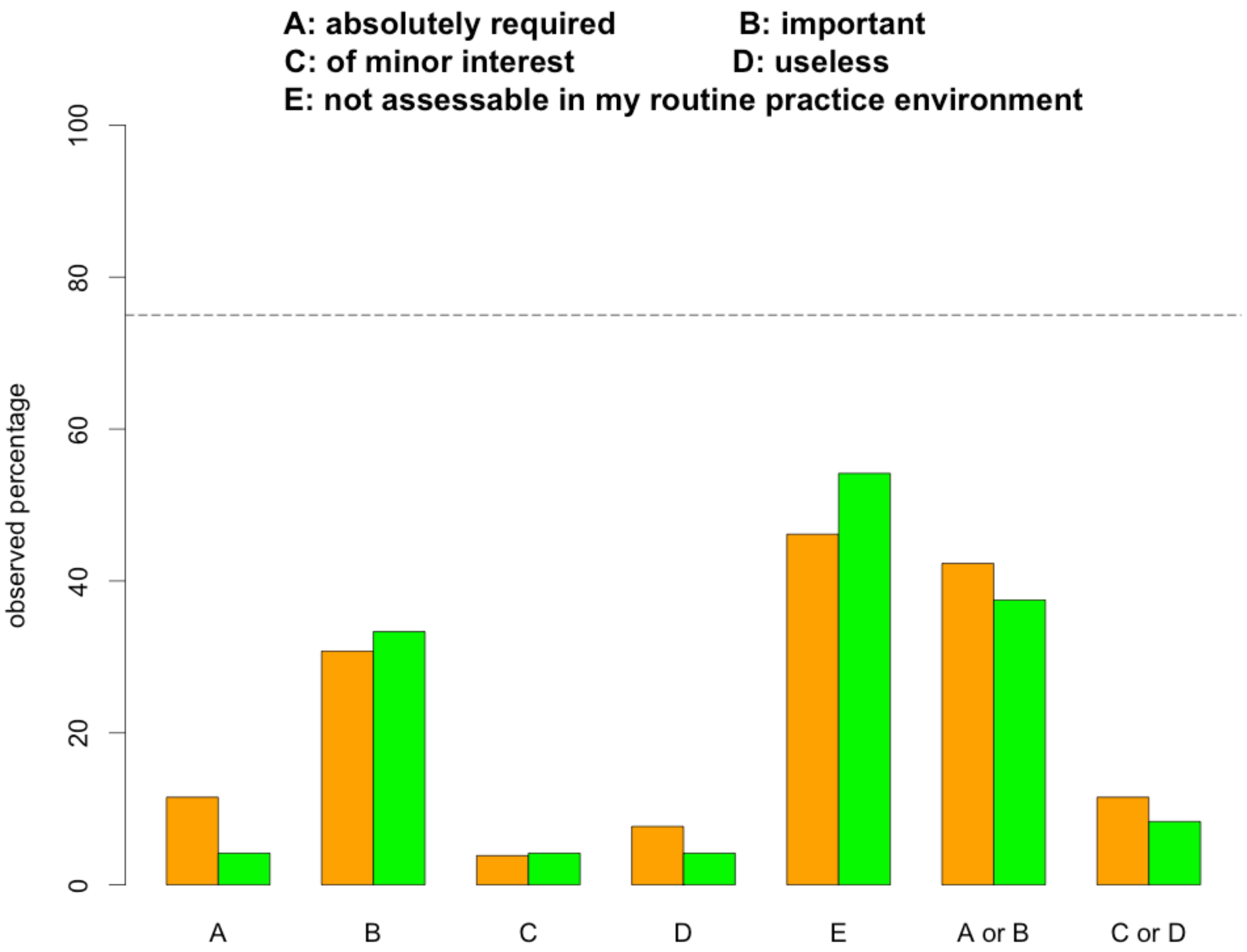

Q21: Natural killer cells.
For the positive diagnosis of reactive hemophagocytic syndrome, a low activity of natural killer cells is:

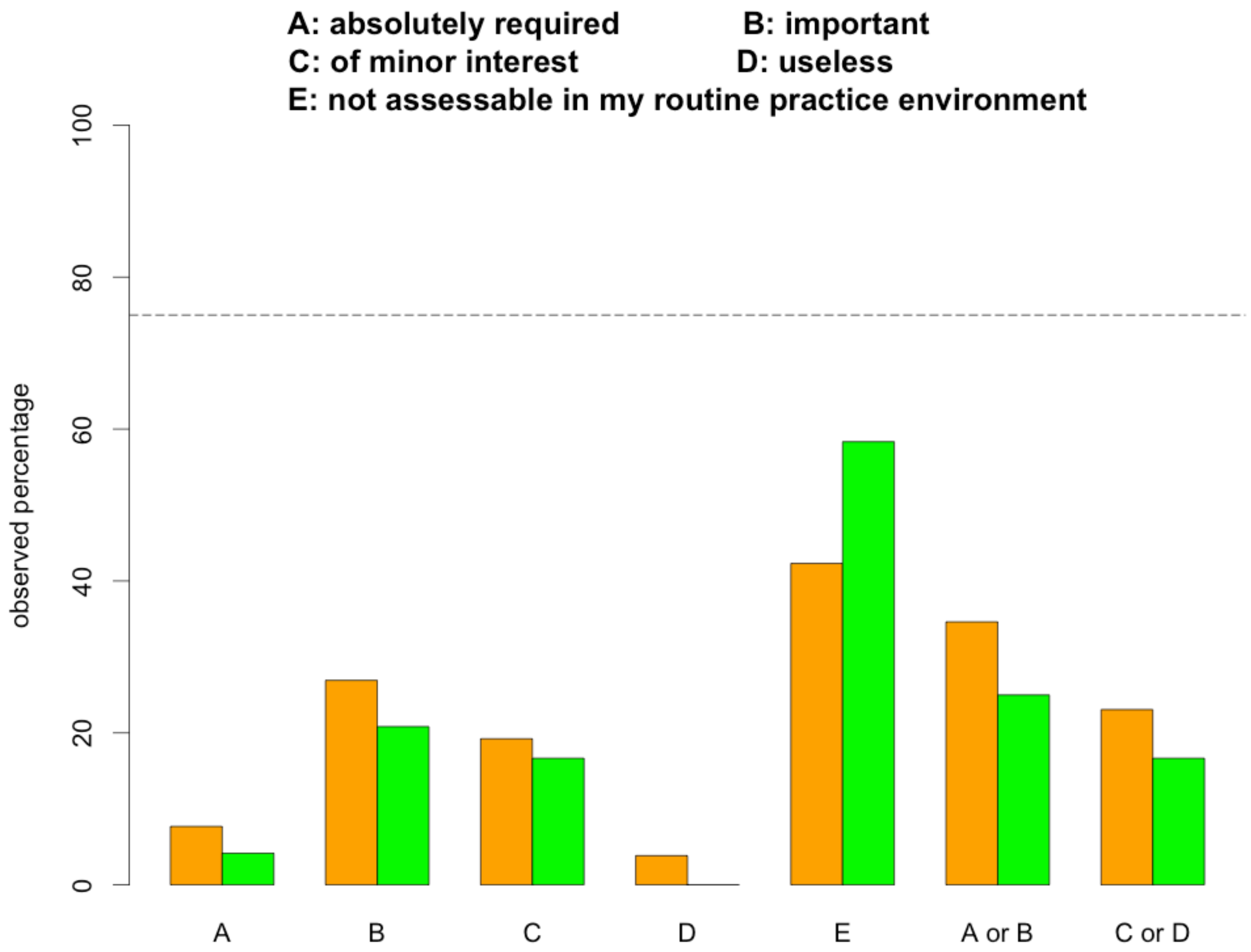

Q22: Serum albumin.
For the positive diagnosis of reactive hemophagocytic syndrome, a low level of serum albumin is:

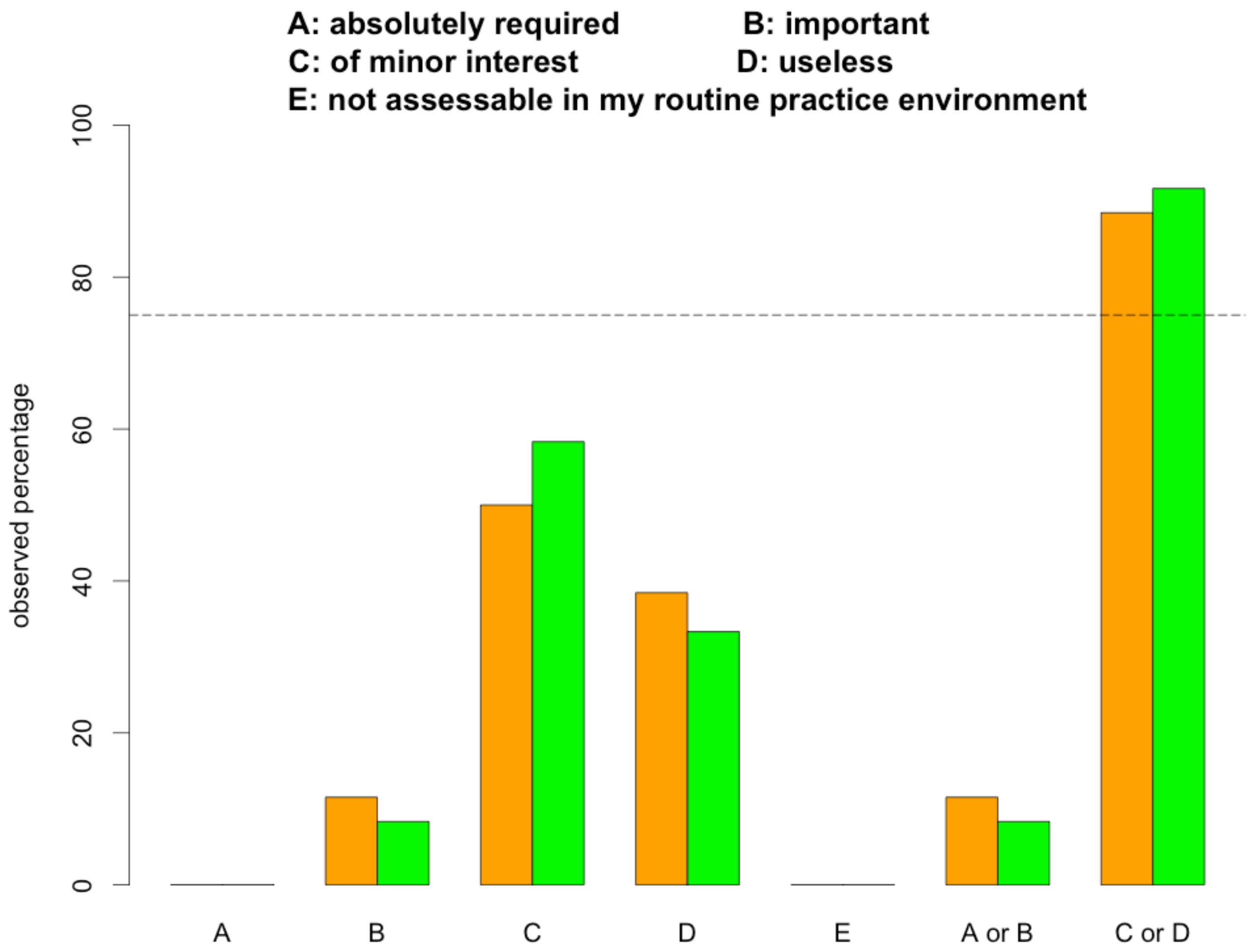

Q23: Activated partial thromboplastin time.

For the positive diagnosis of reactive hemophagocytic syndrome, a short activated partial thromboplastin time is:

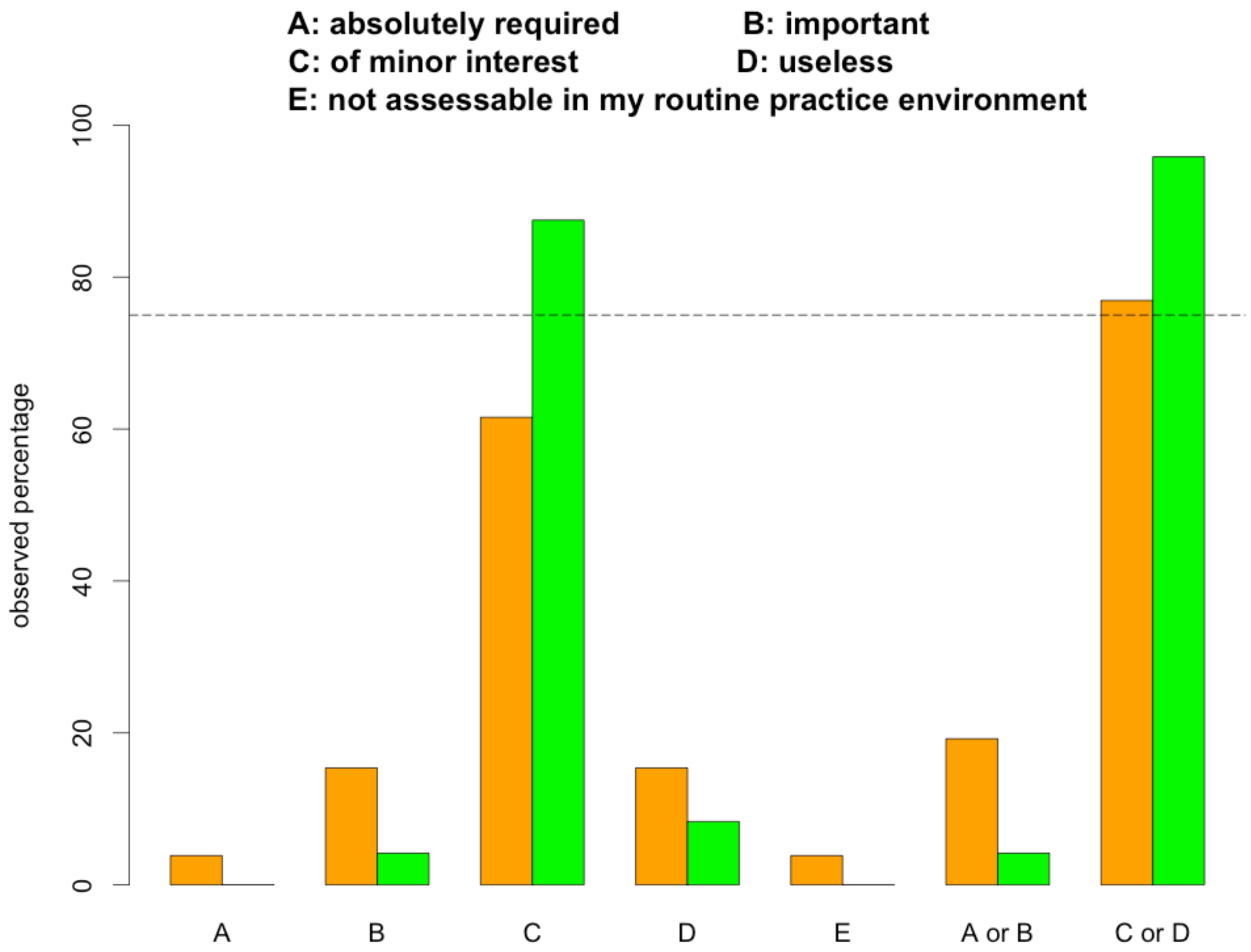

Q24: C-reactive protein.

For the positive diagnosis of reactive hemophagocytic syndrome, a high level of C-reactive protein is:

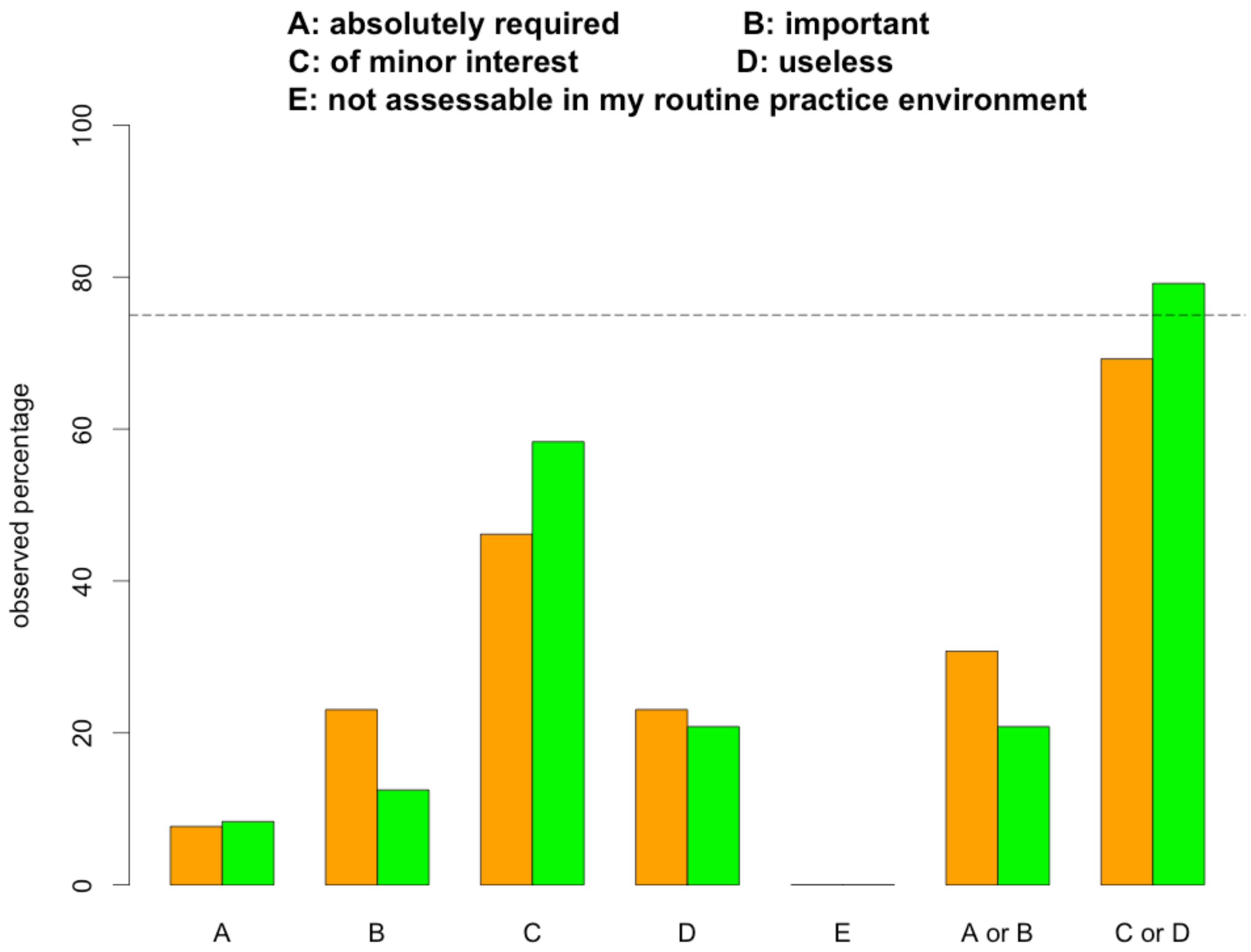

Q25: D-dimer.
For the positive diagnosis of reactive hemophagocytic syndrome, a high level of D-dimer is:

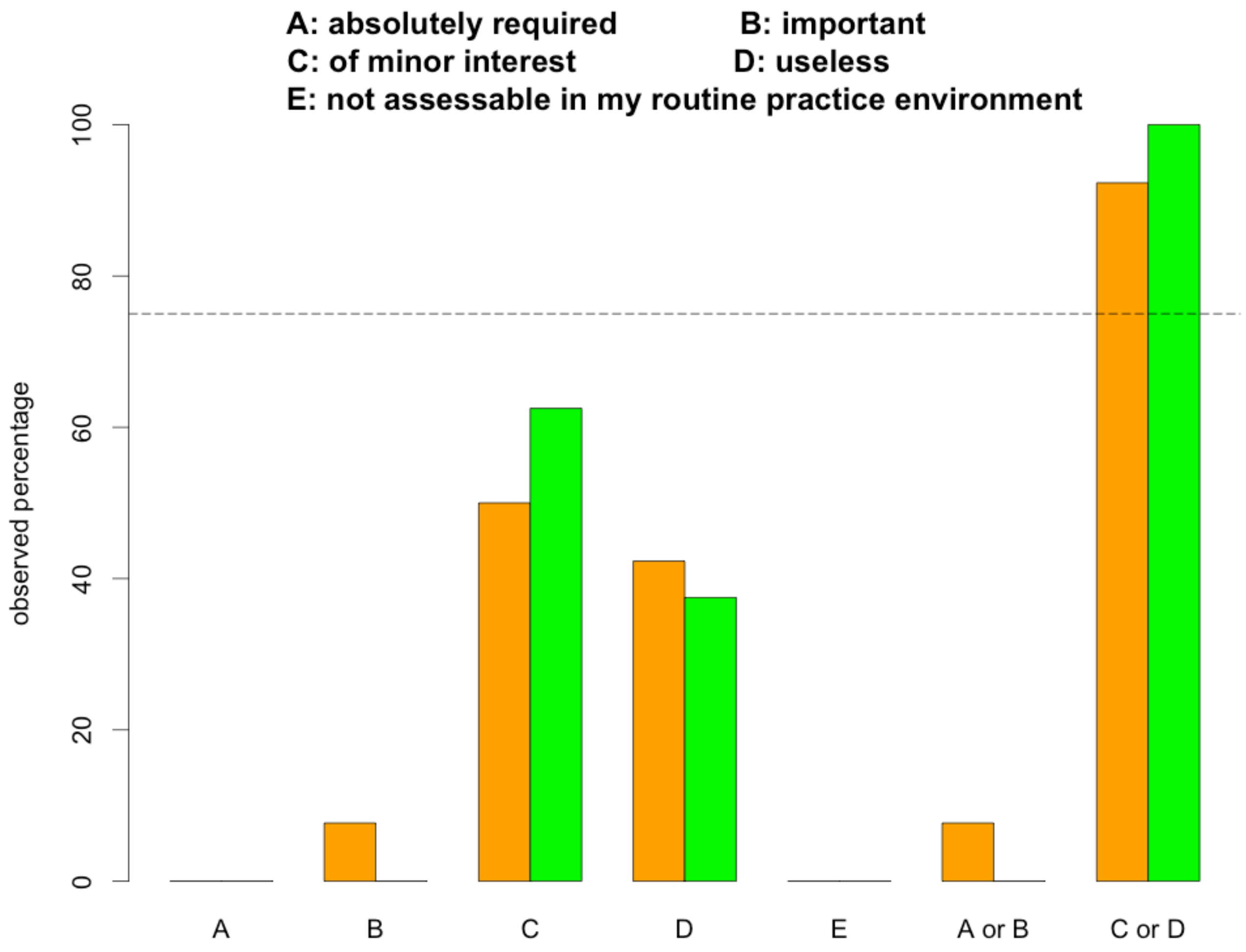

Q26: Hemophagocytosis pictures.
For the positive diagnosis of reactive hemophagocytic syndrome, hemophagocytosis pictures on a bone marrow aspirate or tissue biopsy are:

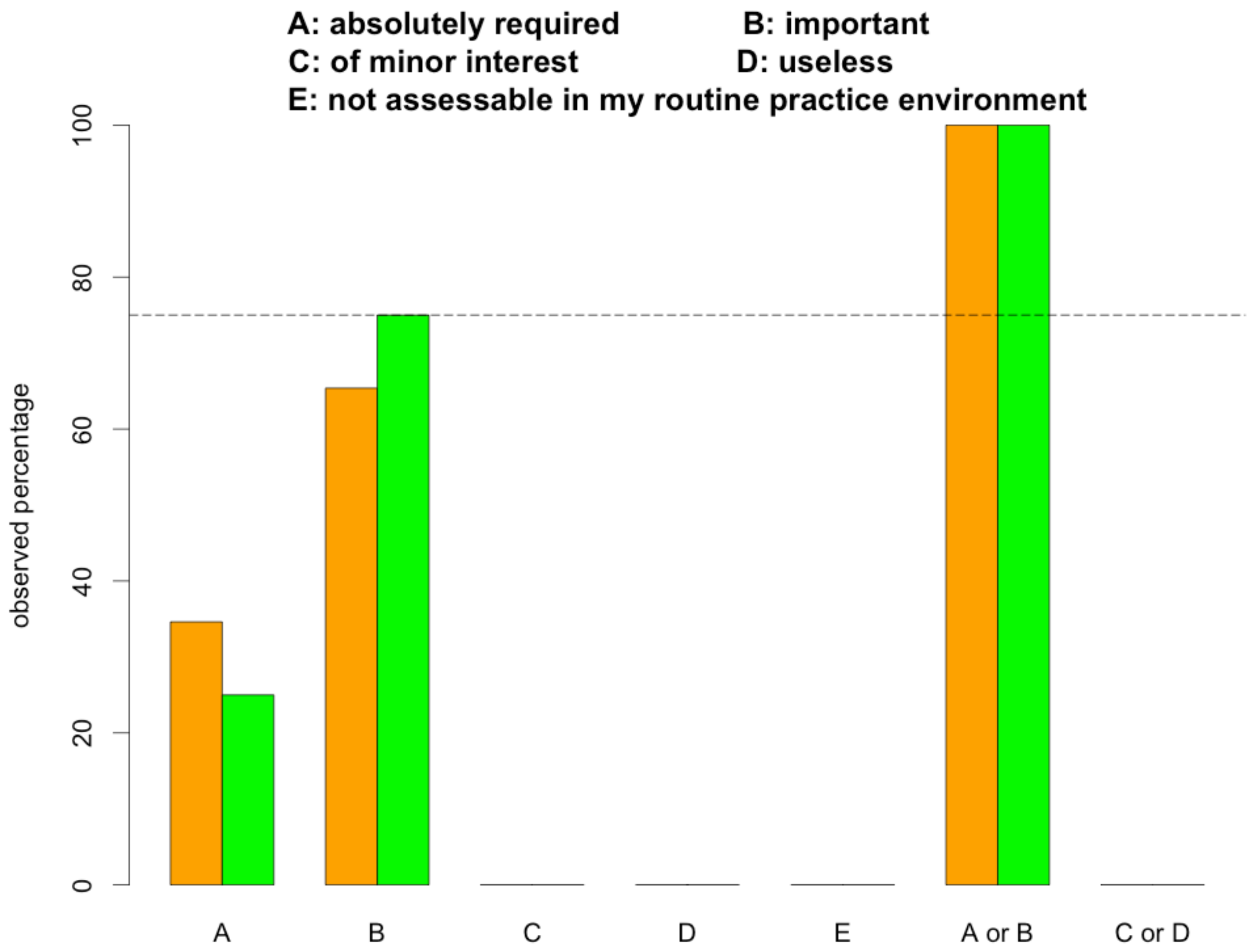

Supplement: Figure S3 — Distribution of the answers at the first and second delphi round, for each of the 26 questionnaire items. (PDF) [file pone.0094024.s003.pdf]
