# Supplementary material for: A Web-Based Delphi Study for Eliciting Helpful Criteria in the Positive Diagnosis of Hemophagocytic Syndrome in Adult Patients
Source: PLoS One. 2014 Apr 7;9(4):e94024. doi: 10.1371/journal.pone.0094024 (PMC3977971; doi:10.1371/journal.pone.0094024)
Supplement: Table S1 — Complete survey data: answers of each participant to the questionnaire at each Delphi round. *Each of the 26 participants is arbitrarily labelled with a number. †Each string composed of 26 letters is a coded pattern representing the whole set of answers of a given expert at a given Delphi round (26 answers): the 26 question wordings are shown in Table 1 of the main manuscript whereas letters A, B, C, D, or E in the string are codes corresponding to the following answers: A, absolutely required; B, important; C, of minor interest; D, useless; E, not assessable in my routine practice environment. ‡Experts #5 and #10 did not participate in the second round. (PDF) [file pone.0094024.s004.pdf]

Expert[*]	Answers at Delphi round 1[†]	Answers at Delphi round 2[†]
1	CBCBCABCADCDDCDBCEEECDDBDB	BBCBCABBADCDDCCBCEEECDDBDB
2	BAABBAAABCBCCBBBCDEECBEDDB	BAABCAABBCBCCCBBBEEEEBCDDB
3	CACBCBBBBCBBCCBBBEEEEDDADB	DACBCBBBACBCCBBBBEEEEDDACB
4	CCDCDABBACBCDDCAAEEEECCCCB	BBCBDABBACBCDDDAAEEEECCCDB
5	BAAACABBBCCCCDDCCBAAADDDDB	‡
6	ABBBCABCAEBCCDBCBBBBADCBCB	BBBBCABCAEBCCDBCBBBBADCCCB
7	BABBCAABABBBCCCBBBABBBBBBB	BABBCABBACBCCCBBBBBBBCCCCB
8	BBCBCABBACBBCDBBBBBEEDCCDB	BBCBCABBACBCCDBBBBBEEDCCDB
9	BBCBCBBBCDBBCCBCCDEEEDCDCB	BBCBCABBBCBCCCBBBEEEECCCCB
10	CBBBBBBBBEBBECBBBBEEECBCCA	‡
11	CBCBCAABABBCCBCABEEECCCBCB	BBCBCABBACBCCBCBBEEECCCBCB
12	CCBBCBBBACCCCCCBCBBBBCCCDA	CBCBCBBBAECCCCCBCBEBBCCCCA
13	ABDDDBBBBDDDDDCDDDDDDDCDCA	ABDDDABBBECDDDCDCDEEEDCDCA
14	BACBCAABABBBCCBBAABABCCBCB	BACBCAABABBCCCBCBAEAECCCCB
15	AABBCAABAECCCBBABEEEECCCDA	BACBCABCAECCDCCACEEEECCDDB
16	CCCCCBCCAECDDDCCBBABCDCCDB	CBCCCBCCACCDDDCCBBABCDCCDB
17	BAABCAAABEBCBBCBBEEEBCCCCB	BBBBCAABAEBBBBBBBEEEECCCCB
18	BACACABBABCDCCBBBBEBEDCDCB	CACBCABBAECCCCBBBEEEECCCCB
19	ABCBCABCAEBCBCBBDEEBEDDDDB	BACBCABBAEBCCDBBCEEBEDCDDB
20	BACBCBBBACBBCCBCBEECECBCDB	BACBCABBACBCCCBCBEECEDCDDB
21	CBBBCBBCABCCCDCCCCBBBCCCDB	BBBBCBBCACCCCDBBCCBBBCCCDB
22	ACCBBAABABBCCDBBCBBBCCBCCA	ACCBCAABABBCCDBBBBBBCCCCCA
23	BABBDDABAECDDBBCBAEABCCCCA	BBBBDAABAECDCBCCBBEBBCCCCB
24	CBCCCAABCCBCCDCDBEEEECCCCA	CBCBCAABCCBCCDCDBEEEECCCCA
25	BBCBDAACACABCCBCBCBDBBAABA	AACBDAACACABCCCCBCBDBBBACA
26	AACBDCBBBEBDDDDDBCEEECCBCA	BACBDABBBEBDCDDDBEEEECCBCA
